# A Matter of Timing: Identifying Significant Multi-Dose Radiotherapy Improvements by Numerical Simulation and Genetic Algorithm Search

**DOI:** 10.1371/journal.pone.0114098

**Published:** 2014-12-02

**Authors:** Simon D. Angus, Monika Joanna Piotrowska

**Affiliations:** 1 Department of Economics, Monash University, Melbourne, Victoria, Australia; 2 Faculty of Mathematics Informatics and Mechanics, Institute of Applied Mathematics and Mechanics, University of Warsaw, Warsaw, Mazowieckie, Poland; Princess Margaret Cancer Centre, Canada

## Abstract

Multi-dose radiotherapy protocols (fraction dose and timing) currently used in the clinic are the product of human selection based on habit, received wisdom, physician experience and intra-day patient timetabling. However, due to combinatorial considerations, the potential treatment protocol space for a given total dose or treatment length is enormous, even for relatively coarse search; well beyond the capacity of traditional *in-vitro* methods. In constrast, high fidelity numerical simulation of tumor development is well suited to the challenge. Building on our previous single-dose numerical simulation model of EMT6/Ro spheroids, a multi-dose irradiation response module is added and calibrated to the effective dose arising from 18 independent multi-dose treatment programs available in the experimental literature. With the developed model a constrained, non-linear, search for better performing cadidate protocols is conducted within the vicinity of two benchmarks by genetic algorithm (GA) techniques. After evaluating less than 0.01% of the potential benchmark protocol space, candidate protocols were identified by the GA which conferred an average of 9.4% (max benefit 16.5%) and 7.1% (13.3%) improvement (reduction) on tumour cell count compared to the two benchmarks, respectively. Noticing that a convergent phenomenon of the top performing protocols was their temporal synchronicity, a further series of numerical experiments was conducted with *periodic* time-gap protocols (10 h to 23 h), leading to the discovery that the performance of the GA search candidates could be replicated by 17–18 h periodic candidates. Further dynamic irradiation-response cell-phase analysis revealed that such periodicity cohered with latent EMT6/Ro cell-phase temporal patterning. Taken together, this study provides powerful evidence towards the hypothesis that even simple inter-fraction timing variations for a given fractional dose program may present a facile, and highly cost-effecitive means of significantly improving clinical efficacy.

## Introduction

Radiotherapy continues to hold a significant place in the treatment of cancer worldwide, with one estimate suggesting that as many as 4 in 10 cancer patients will receive radiotherapy as part of their treatment [1], [2]. Furthermore, radiotherapy is cost-efficient typically accounting for only a fraction of the total cost of treatment [3] with ongoing technological advances dramatically increasing the precision and efficacy of radiotherapy tools [4]. Radiotherapy is commonly applied [5]–[8] as a ‘multi-fraction’ program worked out by the radiotherapy planner consisting of small (1-5 Gy) doses applied with a fixed inter-fraction regime (e.g. once or twice daily) with weekends normally given over to rest. Multi-fraction (low-dose) programs are regarded for their ability to deliver larger total radiation doses without the associated negative impacts on the surrounding healthy tissue that would be associated with an equivalent single-dose protocol. However, if one considers a program (or *protocol*) as a list of time-gaps between fractions where the time-gaps could take any value between 18 h to 30 h in 30 min steps, then even a two-week, once-per-day, ‘10 fraction’ program, could be constructed in any one of over 95 trillion ways 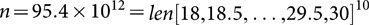. Against this enormous space, clinicians presently utilise a vanishingly small set of ‘standard’ protocols [5]–[8] with the majority utilising 24 h solar and work-place cycles rather than respecting any in-built metabolic or cellular rhythms of the cancerous cells they target.

An obvious question thus arises: could significant therapeutic gains be identified by simply changing the *timing* of multi-fraction programs? Ideally a positive answer to this question would not demand large changes to the existing fractional dose or inter-fraction timing already in use in the clinic so as to stay close to the known impacts of radiotherapy on healthy tissues. Clearly, traditional, *in vitro* (not to mention *in vivo*) techniques face prohibitively high search costs for the kind of search space available [9]. Indeed, a parallel, combinatorial concern, has recently been voiced by workers in combined drug oncology [10] who argued (p.1) that a ‘qualitatively new approach’ to searching the vast space of possible treatment protocols was required.

In this work, we extend our existing, mature, fully calibrated MCS cellular automata model of EMT6/Ro dynamics [11], [12] by adding a calibrated multi-fraction irradiation module. The extended model is then situated within a powerful genetic algorithm (GA) non-linear, constrained, search environment [13],[14] to discover novel candidate protocols that exhibit superior tumour control properties compared to existing benchmarks. We then conduct a *targeted*, systematic, search in the region identified by the GA, which yields quasi-optimal, candidates that confer *significant* and *substantial* improvements in tumour control over presently used, clinical treatment protocols. Significantly, these improvements arise due only to exploiting a single dimension of freedom: the *timing* of the fractions. Subsequent numerical simulation demonstrates the robustness of these findings to a variety of small, but feasible, mistakes in the fractionated timing. Finally, we demonstrate the likely mechanism of the efficacy of the quasi-optimal tumours: the exploitation of underlying cell-line dose-response cell-phase dynamics.

Our work differs substantially from the related work of [15] and [16]. First, we develop a high-fidelity model of EMT6/Ro dynamics under multi-dose irradiation as opposed to a low fidelity theoretical exploration. Second, by exploiting the computational power of the GA search technology, our findings result from *search* as opposed to selective candidate choice. Third, we vary only the *timing* of the fractions, holding all other characteristics to their clinically active (benchmark) levels so as to minimise the likely introduction of any undesirable side-effects beyond those already associated with the benchmark treatments as opposed to varying both dose and timing, or to adding chemo-therapy agents to the mix.

Hence, our constrained search, on a fully calibrated, high-fidelity, *in silico* (tumour dynamics/multi-dose irradiation) system, provides considerable hope for translational outcomes in *in vitro* and ultimately, *in vivo* environments.

## Results

### The Calibrated Multi-Fraction Irradiation Module

The multi-fraction irradiation module added to our existing [11], [12] MCS cellular automata model of EMT6/Ro dynamics had a single control parameter, 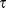 the half-time of reciprocal repair [17] (for details of the module and calibration, see Materials & Methods section). Our calibration strategy for 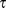 sought to minimise the mean-squared error (MSE) between the effective dose achieved within our extended model as compared to 18 distinct multi-fraction irradiation experiments for EMT6/Ro spheriods as reported in [18] and [19]. The experimental studies provided a robust test of our calibration as together they comprised protocols with fractional-counts ranging from 2 to 5 fractions, and fractional-doses ranging from 4 to 13 Gy.

**Figure 1:**
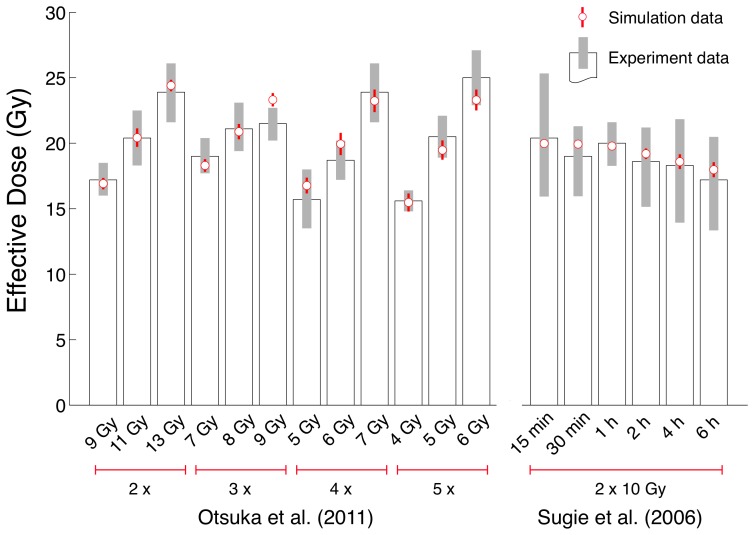
Comparison of calibrated numerical simulation outputs to equivalent experimental data across 18 independent conditions. Simulation data (red open symbols) represents mean effective dose for the model at the optimal fit (minimal MSE) value of 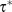, averaged over 10 case library tumours at one random seed (10 replicates). Experimental data taken from [18] and [19] (bars): Otsuka *et al.* 2011, [18], (12 experiments) perform experiments in which the number of fractions and the fractional dose are varied, whilst keeping inter-fraction time-gaps at 4 h; while Sugie *et al*., [19], (6 experiments) use a consistent 2
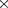
10 Gy protocol, varying the inter-fraction time gap as shown. The grey bars indicate the region of 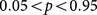 for the experimental data, while the red lines represent the same for the numerical simulations.

It was found that the MSE surface was smooth and single peaked, producing an optimal calibration 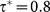, falling within Fowler's [17] experimentally reported values for the half-time of reciprocal repair, for other cell types. In Fig. 1 we present a comparison of our simulation mean effective dose results at the optimal value of 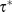 to those of the two reference studies. As can be seen in the figure, the mean simulation results fall within the 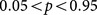 confidence interval for 17 of the 18 experiments, with the single outstanding experiment falling just outside the interval. We are not aware of any other numerical spheroidal model which achieves such close quantitative fit to a key, summary, measure of multi-fraction irradiation application.

### Search by Genetic Algorithm

In all numerical experiments, candidate protocols were scored by comparing the resultant tumour cell count across 10 distinct cases at day 10 (5 days after the end of the treatment period) to the same when one of two benchmark protocols were applied. The 5 day lag after cessation of the treatment period prior to measurement ensures that any re-population phenomena [20] is accounted for in the scoring of candidates. Both benchmarks were constructed to proxy current clinical practice for low-dose, multi-fraction, irraditation treatments [5]–[7]. The characteristics of benchmark I (BMI) and II (BMII) are presented in Table 1. We scored each considered protocol by calculating a one-tailed probability that the candidate produced a significantly improved tumour cell count reduction than the relevant benchmark.

**Table 1:** Irradiation Treatment Protocol BMI and BMII.

Benchmark	Total Fractions	Fractions Per Day	Dose Per Fraction (Gy)	Inter-Fraction Time-delay (h)	Total Dose (Gy)
I	8	2	1.25	(6 h, 18 h)	10
II	5	1	2.00	24 h	10

Benchmark, multi-fraction, 10 Gy, irradiation protocols I and II considered in this study, following active, ‘standard’, clinical protocols as summarised in [5].

In the first phase of candidate search a Genetic Algorithm (GA) [13], [14] was used to efficiently identify search zones within the vast solution space. A total of 1,113 and 1,100 candidates were analysed in benchmark I and II, respectively, comprising over 500 generations. Table 2 presents the results of a detailed study (20 random seeds over all 10 case library tumours: 200 replicates per protocol) of the top 3 protocols in each benchmark region as discovered by the GA methodology. Within the region of BMI, the top ranked candidate led to an average cell-count reduction of 9.4% (range: 3.9–16.5%). For BMII, the top ranked protocol yielded a 7.1% reduction in cell count on average (range: 2.5–13.3%). In terms of significance, each top performing protocol yieled mean *p-value* comparisons with the relevant benchmark population of 0.039 and 0.069, respectively, satisfying a 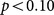 one-tailed significance test of an improvement in both cases.

**Table 2:** Top 3 Performing Protocols Discovered by GA Search.

Rank	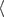 *p*(*ρ_i_*) 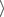	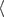 *S_i_* 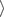 (range) (%)	GA.Inh	*ρ_i_* (h)
BMI				
1.	0.039	9.4 (3.9–16.5)	tmnt	{ 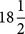 , 20, 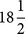 , 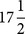 , 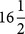 , 13, 10}
2.	0.112	8.1 (−0.2–13.7)	top1	{ 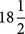 , 20, 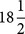 , 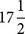 , 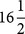 , 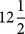 , 10}
3.	0.142	7.0 (−6.4–17.9)	tmnt	{ 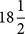 , 20, 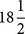 , 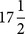 , 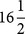 , 14, 10}
BMII				
1.	0.069	7.1 (2.5–13.3)	top5	{15, 22, 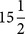 , 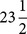 }
2.	0.317	4.6 (−3.5–15.8)	top5	{15, 19, 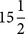 , 18}
3.	0.376	4.0 (−4.6–16.5)	top1	{15, 23, 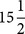 , 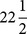 }

Best performing protocols, ranked by *p*(*ρ_i_*), are given with summary statistics: 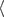 *S_i_* 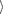 average % (and range of) cell-count reduction (relative to the given benchmark); and the inheritance operator which produced the protocol (top5, ‘Top 5’; tmnt, ‘Tournament’; top1, ‘Top-1’). Data represent in-depth study of each candidate by applying it to all 10 tumours of the case library, under 20 independent random seeds.

### Synchronicity

Upon inspection of the resultant library of candidates created and tested through the GA search, it became apparent that there was a tendency for the more successful protocols to exhibit a smaller distribution of inter-fraction time-gaps. That is, as the GA search progressed, the pool of novel candidates appeared to exhibit *quasi-synchronicity* around a given 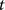. This tendency is evidenced by the top ranked candidates presented in Table 2, where of the 33 time-gaps presented, 23 (70%) of them fall within the range 15 h to 20 h.

**Figure 2:**
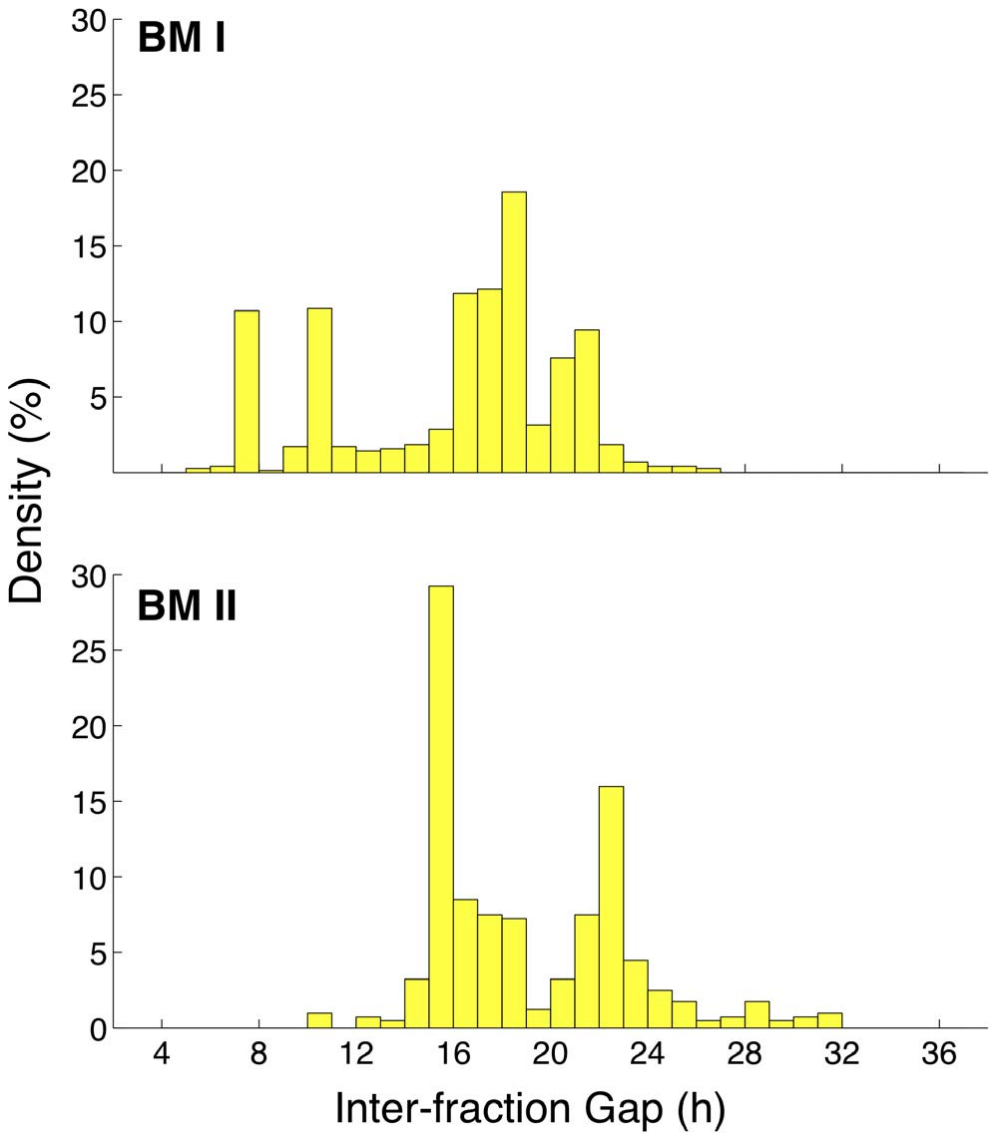
Observed inter-fraction gap synchronicity in more successful candidate protocols discovered by GA search. Density plots of the inter-fraction time-gap (h) for the pooled time-gaps present in the top 100 protocols (ranked by 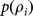) for BMI (top) and BMII (bottom). Note: the inter-gap fractions are aggregated into bins of size 1 h.

To explore this phenomenon, density plots of pooled inter-fraction time-gaps from the top 100 candidates (ranked by 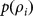, where 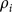 denotes the *i*th irradiation treatment protocol, see Materials & Methods section) for each benchmark region were plotted (see Fig. 2). From these data, it was found that for both BMI and BMII half of the time-gaps fell in the region 14 h to 19 h (47.4% and 55.6%, respectively for each benchmark) with the modal time-gap in each benchmark being 18.5 h and 15.5 h, respectively.

Next, following the indications of these results, targeted, *periodic* time-gap candidate experiments were conducted. Specifically, hand-crafted candidates having periodic time-gaps within the range 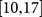 h and 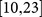 h were applied to the tumour case library as before for benchmark I and II, respectively. To provide as rigorous a test as possible, 10, 20 or 40 distinct random seeds were used for each of the 10 tumours in the case library. Note, the upper bound for BMI must not exceed 17 h since beyond this point, the total protocol treatment period would be greater than 5 days, with over-flowing fractions being dropped (disallowed) by the irradiation module.

**Figure 3:**
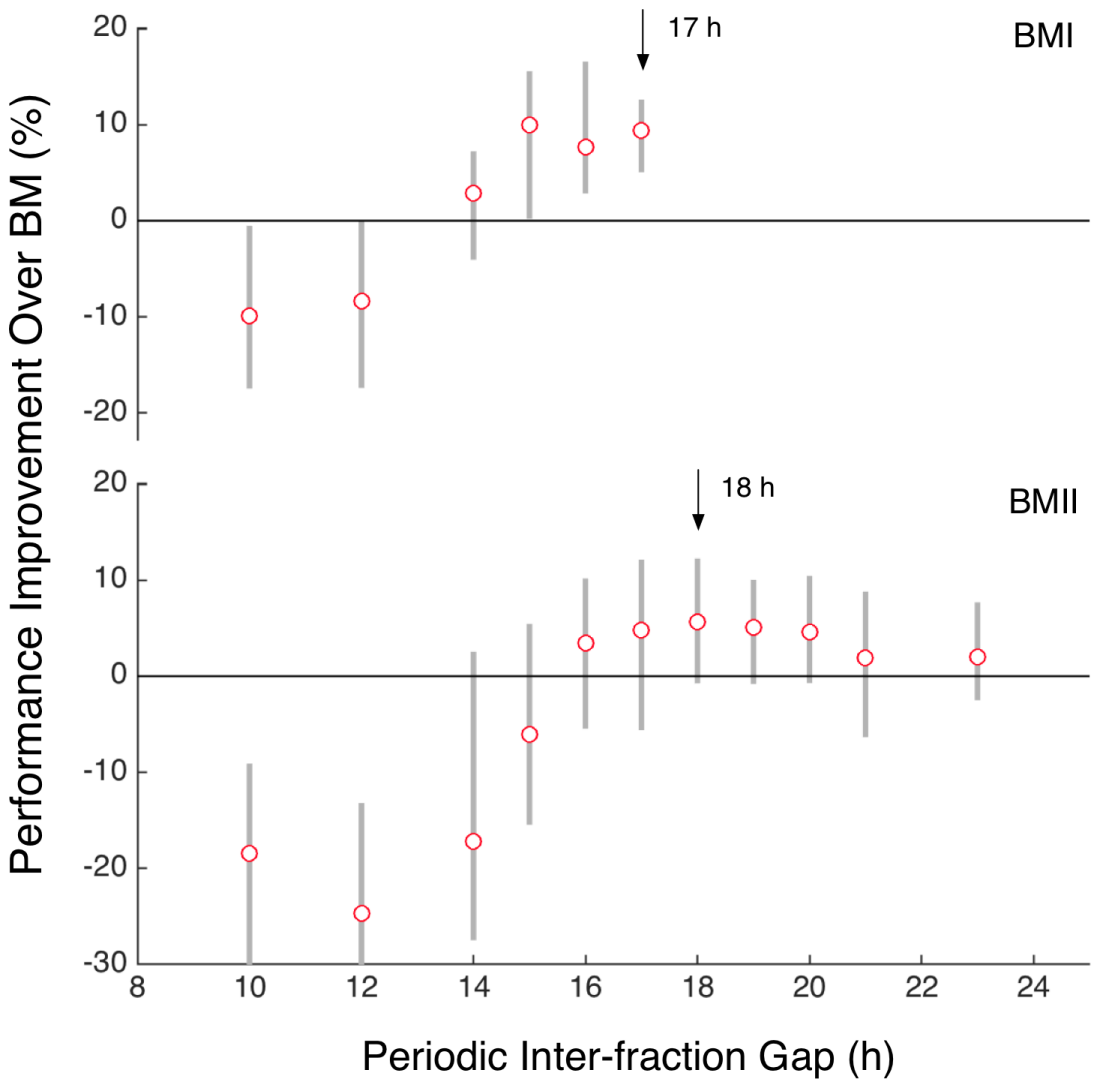
Summary performance improvements of periodic candidates tested in each benchmark condition. Grey bars indicate the range (min – max) of average normalised cell count difference to the given benchmark protocol across the 10 tumours in 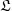. Markers indicate the average. Quasi-optimal periodic candidates indicated by arrows. Note that a varying number of random seeds (from 10 to 40) were employed at each periodicity level. See Table 3 for details.

Table 3 and Fig. 3 present the results of these experiments. As expected, in each benchmark, the top performing periodic candidate fell within the high-density band indicated by the GA search phase. For BMI, we consider that the top performing, periodic, candidate was the 17 h version. Whilst strictly speaking the 17 h protocol did not demonstrate the best *mean* performance it nonetheless yielded the strongest overall performance with a significantly higher level of minimum outcome and statistical significance than other protocols with an average normalised cell fraction improvement of 9.4% (range: 5.3–12.3%), at average 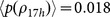 (one-tailed). Compared to the top-performing protocol from the GA search (refer Table 2) the periodic 17 h candidate yielded a similar benefit, but with higher statistical significance (rank 1 GA BMI candidate 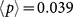). For BMII, the best performing candidate was found to be the periodic 18 h protocol which produced an average benefit of 5.6% (range: −0.5–12.0%) at an average 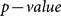 of 0.181. Whilst not reaching the same level of statistical significance, the average and range of improvement of the periodic 18 h protocol was found to be similar to that of the best GA protocol in BMII.

**Table 3:** Performance of periodic candidates under each BM context.

*ρ_i_* (h)	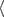 *S_i_* 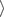 (%)	(range)	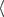 *p*(*ρ_i_*) 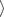	(s.d.)	seeds
BMI					
{10, …, 10}:	−9.9	(−17.2– −0.8)	0.926	(0.123)	10
{12, …, 12}:	−8.3	(−17.1– −0.4)	0.889	(0.155)	10
{14, …, 14}:	2.9	(−3.8–6.9)	0.263	(0.292)	20
{15, …, 15}:	10.0	(0.5–15.3)	0.056	(0.139)	40
{16, …, 16}:	7.6	(3.1–16.3)	0.058	(0.075)	40
{17, …, 17}:	9.4	(5.3–12.3)	0.018	(0.025)	40 *
BMII					
{10, …, 10}:	−18.4	(−29.8– −9.4)	0.997	(0.005)	10
{12, …, 12}:	−24.7	(−38.3– −13.5)	1.000	(0.001)	10
{14, …, 14}:	−17.2	(−27.2–2.3)	0.917	(0.235)	10
{15, …, 15}:	−6.0	(−15.2–5.2)	0.730	(0.398)	10
{16, …, 16}:	3.4	(−5.2–9.9)	0.263	(0.335)	20
{17, …, 17}:	4.8	(−5.3–11.8)	0.192	(0.326)	40
{18, …, 18}:	5.6	(−0.5–12.0)	0.181	(0.229)	40 *
{19, …, 19}:	5.1	(−0.5–9.7)	0.133	(0.216)	40
{20, …, 20}:	4.6	(−0.4–10.2)	0.150	(0.197)	40
{21, …, 21}:	1.9	(−6.1–8.5)	0.413	(0.427)	40
{23, …, 23}:	2.0	(−2.2–7.4)	0.345	(0.276)	20

Periodic (constant interfraction time-gap) candidate performance by average one-tailed p-value score and mean cell count reduction (with range). Maximal performance of 17 h and 18 h periodic candidates in BMI and BMII contexts respectively indicated by ‘*’. The last column indicates the number of independent random seeds used to construct the summary statistics.

### Robustness

If periodic time-gap candidates present quasi-optimal performance with great simplicity, how important is their *exact* periodicity? This is a question of *robustness* and has critical importance for the potential therapeutic benefits of the periodic protocols: if the power of the periodic candidates requires the patient to receive each fraction within a 30 min (or smaller) window, though powerful, the periodic approach loses considerable practicality.

To probe the robustness of the periodic 17 h and 18 h candidates, a series of ‘tremble’ experiments was conducted. Specifically, 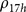 and 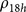 were perturbed by randomly switching one or more time-gap with another time-gap drawn from 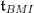 or 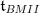 for the two benchmark regimes BMI and BMII, respectively, where 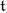 denotes the vector of wait-times between fractions within the given irradiation protocol. In order that small to large deviations from periodicity could be studied, perturbed candidates having up to a fixed total deviation from the quasi-optimal periodic candidate were considered. For example, at a total tremble time of 4 h, two 17 h elements of 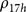 might be replaced with 16 h and 14 h, respectively, which collectively provide 4 h of deviation from 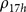. For a patient who is scheduled to receive the 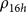 protocol, this would be equivalent to receiving two of the eight fractions over the first five days of treatment 1 h earlier and 3 h earlier, respectively, than the prescribed schedule. For BMI and BMII total deviations away from 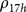 and 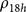 amounting to 2, 4, and 8 h were studied. In all, ten distinct candidates were developed, randomly, at each total deviation level, and applied to the entire (ten tumour) case library (i.e. 100 trials *per* total deviation experimental). Note, each deviation candidate was checked to ensure it complied with the 5 day total treatment time limit.

For BMI, it was found that the average cell reduction performance fell by less than 1.5% even with a total deviation from 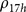 of 8 h (equivalent to all 8 fractions being administered up to an hour away from their scheduled time). Indeed, the worst cell reduction benefit of the 8 h (total) perturbed protocols was still 1.3% better than BMI with the best 8 h (total) perturbed protocol conferring a remarkable 12.6% benefit. For BMII, trembles were found to be slightly more significant, with the 8 h treatment leading to an average drop in performance of 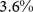 cell reduction when compared to 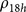. Nevertheless, the 4 h treatment resulted in just a 1.1% drop in performance on average.

## Discussion

The main aim of this work was to explore numerically the combinatorially vast multi-fraction treatment protocol space which exists around clinically active multi-fraction irradiation protocols. Specifically, it was hoped that significant and substantial tumour control performance gains could be gleaned by making only small, but important, changes to existing protocols, thereby giving any performance enhancing protocols so discovered the best chance of being applicable in the clinic – the ultimate performance theatre. Indeed, if it could be shown that significant and worthwhile benefits could be obtained in a high-fidelity numerical simulation model within a heavily constrained search zone, we believed this would constitute strong evidence that search within a wider, though potentially less comprehensible, search zone (e.g. timing *and* dose) was well warranted.

To accomplish our aim, to our existing, calibrated, model of EMT6/Ro spheroid growth and single-dose irradiation response [11], [12], we have added and re-calibrated a multi-dose (multi-fraction) irradiation response module. After compiling a 10-tumour case study library, two benchmark (both with total does of 10 Gy, and treatment duration of 5 days, or 1 week) multi-fraction protocols were applied multiple times to each tumour in the case library to establish a baseline performance. The extended, multi-fraction, model was then placed within a GA framework to conduct a coarse-grained, constrained, search for performance improving protocols.

It was found that a convergent feature of the top performing protocols discovered by the GA was *temporal periodicity*, i.e. the inter-fraction time-gaps converged, in the best protocols, to a small fraction of the available time-gap space. Probing further, we identified a set of quasi-optimal protocols which each comprised constant inter-fraction time-gaps of approx. 17–18 h and resulted in significant (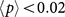 and 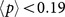, respectively) average tumour size reductions of 9.4% (range 5.3%–12.3%) and 5.6% (range −0.5%–12.0%) over the two benchmark protocols, respectively.

Furthermore, these gains were achieved whilst holding a great number of other characteristics of the protocols at the same levels as the clinically applied, benchmark protocols: the *total dose* over a week-long period; the *number* of fractions administered; and the *quantum* of each fractional dose. The only degree of freedom we exploit is the *timing* of the fractions within a restricted 5-day treatment period. Further simulations showed that tumour control performance improvements do not depend in a knife-edge way on the *exact* periodicity of the protocols: aggregate movements away from exact periodicity of up to 4 h over a week (e.g. administering a fraction earlier or later than prescribed by 0-1 h, four times during a week) result in an average reduction in improvement of less than 1.1%.

Whilst the performance gains identified by the present study may appear modest (up to 12% in each benchmark depending on the tumour), it should be remembered that our study considers only a relatively short, *first week*, of treatment. Furthermore, our methodology allowed for a *five-day* (no irradiation) re-growth period prior to performance measurement, meaning that performance gains are most likely to be material, lasting, improvements (rather than, for example, due to some particular timing of the last fraction or other confounding effect). Typically, a clinical treatment program will deliver 30–50 Gy [5] at the fractionated timetable considered in our work, i.e. a total treatment period three to five times longer than that which we have considered here. Whilst further, long-run, simulations would be needed to verify any conclusions, it seems reasonable to expect that the *cumulative benefit* of the quasi-optimal protocols discovered in our study, if applied over a three or five week period, could be considerably more than 10%, possibly in the region of 20% to 40%.

In summary, the principal result of this paper is that robust, significant, and substantial performance improvements likely lie in the near vicinity of existing clinically active multi-fraction irradiation protocols. Granted, following the prescription of this study, patients may have to receive several fractions out-of-hours during the treatment cycle, but, importantly, the protocols likely introduce no new or significantly harmful side-effects on normal tissues. To our initial question of asking if significant gains are likely possible within a highly constrained search zone on a high-fidelity numerical model, we conclude in the affirmative; giving considerable hope for even greater clinical benefits if one relaxes one or more further degrees of freedom in protocol construction.

Placing these results in the existing computational literature is not easy: whilst there has been many advances in the delivery technology of radiotherapy [3], [4], [21] similar advances in treatment protocols appear to have received less attention. The obvious reason for this imbalance is the far greater costs of trialling many treatment protocols under *in vivo* or in *in vitro* conditions, and, the high level of complexity in developing multi-scaled, high-fidelity, computational models of tumour dynamics under multi-fraction irradiation. The recent computational models of Kempf *et al.* [15] and Powathil et al. [16] are perhaps the closest we are aware of to our work, however, neither provides a quantitative calibration of their model so quantitative comparisons of results are not possible. Similarly, neither present results of *search* for quasi-optimal (or optimal) protocols, instead, presenting the results of given, hand-crafted alternatives to existing clinical protocols. Nevertheless, both works conclude that their tentative results encourage further, more detailed, consideration of irradiation timing especially as it relates to cell-phase cycle considerations. On the other hand, the studies we are aware of which conduct constrained numerical optimisation over irradiation and/or irradiation/chemotherapy solutions [22], [23], find the potential for significant tumour control performance improvements over existing clinical approaches. For instance, Wein *et al.*'s study [22], which holds fractional timing constant and varies the size of each fraction finds an improvement of around 0.20 in tumour control probability (TCP) for comparable (10 Gy/week, 1 or 2 fraction per day) protocols.

Whilst the results of our work cohere with the small, existing, computational literature on the benefits of cell-phase synchronicity in cancer treatments [15], [16], it must be stressed that our approach differs substantially from previous authors in the level of fidelity our model achieves, and thus, the degree of confidence that might be carried from periodic candidates identified in this work to further experiments *in vitro* or *in vivo*. For example, Kempf *et al.* [15], [24] estimate potential radio-sensitivity ‘windows’ due to cell-cycle synchronisation resulting from multi-fraction irradiation protocols. However, whilst importing some parameters from the experimental EMT6/Ro literature, validation of the mechanisms of the basic (no irradiation) model proceeds only at the dynamics of the total-cell count level, with no validation undertaken of the tumour's other properties (e.g. volume, necrotic core dimensions, cell metabolism, cell-phase dynamics), let alone the tumour's response to single- or multi- dose irradiation. At a higher level of abstraction, Powathil *et al.* [16] construct a multi-scale system capable of studying combined chemo-/radiation- protocols within a computational, mathematical model. By way of validation, the authors present a comparison between model cell-phase distributions after a single dose (3 Gy) of irradiation with those of an experimental study. However, no quantitative comparison is made, nor are the model outputs compared to multi-dose irradiation experiments, a limitation the authors note (p.10). Similarly, the work of Alfonso *et al.* [25] which although not considering synchronicity directly, presents a related multi-scale computational spheroid model to study the potential for spatially heterogeneous radiotherapy delivery, includes no external validation. Moreover, in each of these related studies, and many like them [7], [22], [26]–[29], cellular irradiation sensitivity is modeled by employing the Linear-Quadratic (LQ) parameters taken directly from experimental studies; an approach which we have shown in an earlier work [12] can dramatically under-estimate the true probability of cell death due to irradiation, after cell-repair is taken into account.

In contrast, our previous [11], [12] and present works have relied strongly on calibration to a variety of experimental evidence for a single cell line. Not only is our work calibrated to bulk direct- and calculated- (fitted) tumour properties such as cell count, volume, saturation cell count and saturation volume, but also to internal dynamic properties such as the onset of necrosis, the thickness of the viable cell rim, and the progression of the cell-phases over time. Furthermore, we have taken care to rigorously test our model of irradiation response under single- [12] and multi- dose (this work) irradiation. Since the focus of the present work is on the *dynamic*, and *complex*, interaction between (at least) cell-metabolism, cell-phase cycling, mitosis, irradiation damage and death, and cellular repair, we contend that without *multi-dimensional* calibration and validation at each milestone in a model's development, any subsequent results can be considered tentative at best. Subsequently, the results of this paper should be read as complimentary to the foregoing *in silico* and theoretical works on multi-fraction irradiation optimisation, adding considerable weight to previous indications that cellular synchronicity provides a potentially low cost, yet powerful, tool to enhance clinical outcomes.

**Figure 4:**
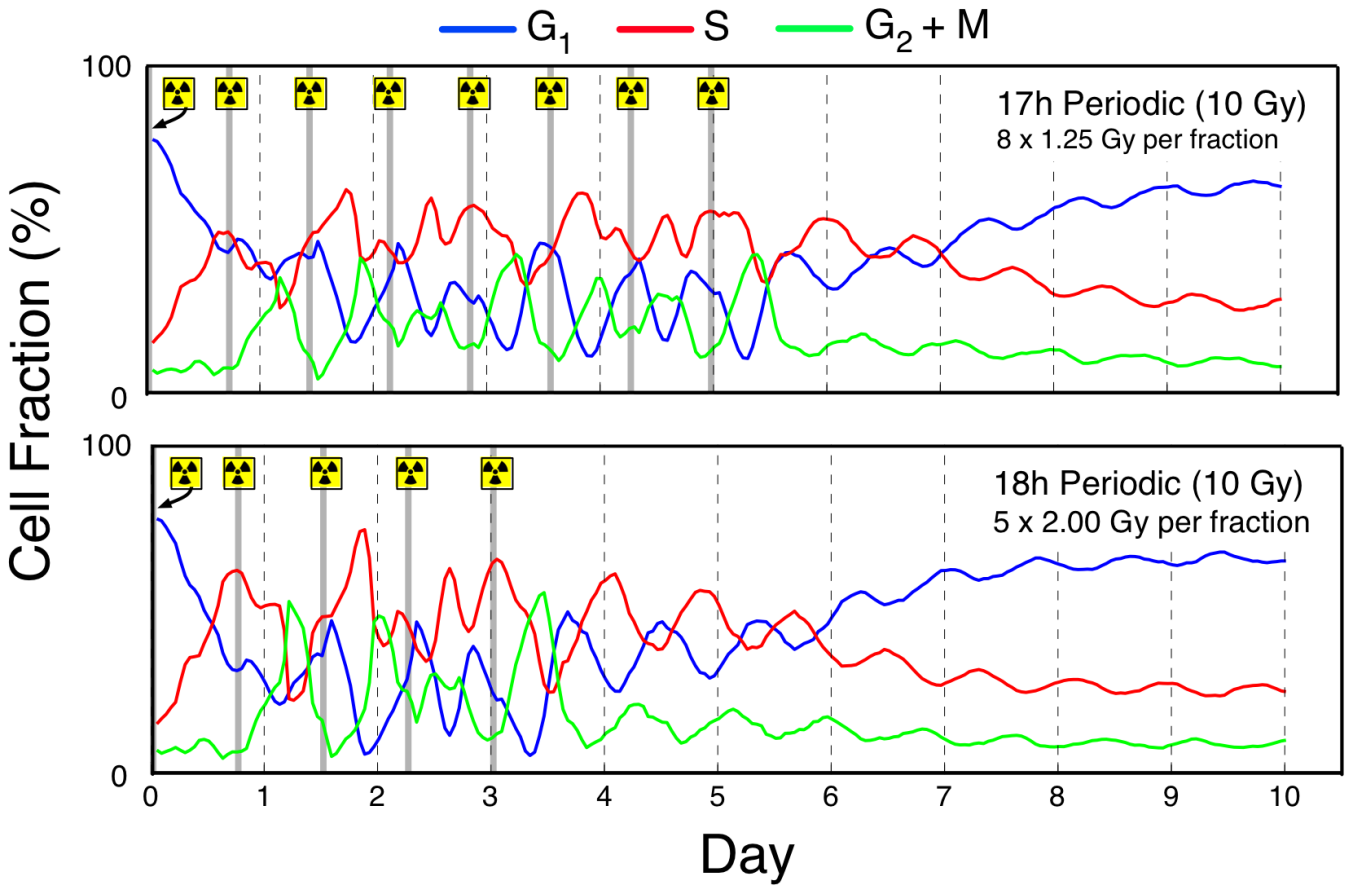
Dynamic cell-phase response of the model under periodic (17 h or 18 h), multi-dose irradiation. 1.25 Gy (BMI) context (top); and 2.00 Gy (BMII) context (bottom). Irradiation symbols and grey lines indicate timing of irradiation fractions. Each cell phase line represents the hourly mean of the cell dynamics traces across each of the 10 tumours in the tumour case library and over 10 unique random seeds (100 replicates in all).

As to the mechanism of efficacy of the 17–18 h periodic protocols discovered by this work, we offer only speculative indications. Clearly, one would suspect that, in line with previous contributions [15], [16], [23] the mechanistic locus would lie in the interaction between the timing of the irradiation fractions and the cell-phase dynamics of the developing tumour. In Fig. 4 we present the mean cell-phase dynamics over the 10 tumours of the tumour case library during, and after, the application of the 17 h or 18 h periodic protocol within each of the benchmark contexts, respectively. Unfortunately, it is impossible to validate these traces with experimental data. To the best of our knowledge cell-phase dynamics in response to *multi-dose* irradiation are available only for V79 Hamster cells [30] under 1.5, 3 or 6 Gy over 12 h of response, and for *single-dose* irradiation of HeLa cells (3 Gy) [16], or EMT6/Ro cells [31] under 3 or 6 Gy. Clearly, given that different cell lines have substantially different cell-phase periods, it is not possible to compare our model with other cell line results. Nevertheless, our model provides quantitatively similar dynamics when a single dose of 6 Gy is applied as in the EMT6/Ro study mentioned (see File S1), so we draw some confidence that the cell-phase response dynamics of the simulations provides a reasonable indication of reality. Figure 4 demonstrates that in both benchmark contexts, the irradiation fractions *create* and then *coincide* with significant periodicity in both the 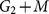 and 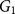 cell-phase fraction time-series. A direct comparison of the cell-phase dynamics of Fig. 4 and the cell-phase dynamics of the two benchmark treatments (see Fig. S5 in File S1) reveals that the coincident periodicity of the 17–18 h protocols are potentially the *key* distinguishing factor which confers its performance benefit; the benchmark protocols routinely ‘miss’ both the 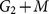 troughs and 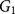 cell-phase peaks. Indeed, calculations (not shown) based on the trough dynamics of the post-irradiation region in Fig. 4 gives rise to a mean 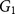 period of 18.3 h in both the BMI and BMII contexts, whilst the 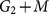 periodic is 22.0 h and 20.3 h, respectively. Since these calculations come from the out-of-treatment region, one could conclude that these measurements reflect the underlying (latent) cell-phase periodicity of the EMT6/Ro cell line. The obvious suggestion of these measurements is that by focussing attention on the 17–18 h periodic protocol region, the natural ‘mode’ that the GA Search and periodic numerical simulations have uncovered, is that of the 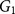 periodicity. These data suggest that the potentially significant and substantial tumour control benefit which appears available to the patient through optimised dose-timing could be obtained with a basic understanding of the cell-phase dynamics of the cancerous cell-line in question. A possible pathway for clinical application would be to biopsy the tumour mass of focus, stabilise in a series of suitable nutrient matrices, apply a basic coarse-grained multi-fraction protocol to the mass (e.g. as BMII) and then study the induced 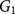 cell fraction peak post-irradiation (as in Fig. 4 or Fig. S5 of File S1) to suggest a likely periodic protocol target. However, such an approach is speculative only until the 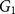 latent cycle peak conjecture can be established across multiple cell lines.

Whilst the present study has attempted to produce a high-fidelity model of EMT6/Ro MCS growth and dynamics under multi-fraction irradiation, several limitations should be acknowledged. First, despite the fact that one of the key drivers in heterogeneous radiotherapy technology advance is the maximisation of radiation delivered to the tumour mass whilst limiting the impact on healthy tissues [3], [21], such considerations are not implemented here. To this we make a few comments: a) that, in principle, adding heterogeneous irradiation beam types and spatial delivery to our model, would be relatively straight-forward, but calibration of this module without published, high-granularity data, seems (at present) highly unlikely; and b) that one of the chief reasons for reducing the scope of search merely to the *timing* of the fractions in a narrow, relatively coarse granularity, band, was to minimise the distance between the side-effects of any discovered candidate protocol and the in-use protocol it departed from. Nevertheless, without experimental data arising from an *in vivo* trial of the periodic protocols proposed in this paper, it is impossible to conclude that negative side-effects on healthy tissues would not arise. Indeed, of the small number of clincial, randomised, trials we are aware of which have considered minor variations to the fractionation schedule [32], [33], adverse (though apparently reversible) morbidity and localised toxicity have been observed in some cases, justifying an approach of cautious optimism.

Second, the periodic candidate protocols arising from the present study cannot be considered anything but ‘quasi’-optimal since in fact, only a vanishingly small fraction of the candidate protocol space was searched by the GA phase of the search methodology. With sufficient compute access or code optimisation, one could either run the GA for many more generations, or conduct a more ‘brute force’ study of the landscape, perhaps sampling at a systematic rate of one candidate per thousand or hundred thousand variants. We will leave such work for other authors to consider. Here, we simply note that GAs are well suited to focussing attention on powerful solution sub-spaces (if not *optimal*) in a variety of difficult and vast solution landscapes [2], [13], [14], [34]–[36].

Third, what the present study gains in fidelity and quantifiability by virtue of its strong adhearance to experimental data available for a single cell line (EMT6/Ro) it lacks in potential generalisability of its results. To take the inferences of this study further, one would need to apply similar calibration techniques to a second, or third cell line and re-discover any tendency towards periodicity in quasi-optimal protocols as found here. At present, we, like all related authors in this research area, are aware of only the excellent data available for the EMT6/Ro cell line across basic tumour dynamics (including progression of the cell phases over time) [37], [38], single-dose irradiation [39], [40], and multi-fraction programs [18], [19]. According to our knowledge no experimental data on any other cell line appears to come close to the EMT6/Ro library at present. However, we expect that with the declining costs of both *in vitro* and *in vivo* laboratory trials, and the gathering momentum of *in silico* multi-scale computational oncology models, this situation will likely change in the near future.

Finally, a limitation which stands for all studies built on non-human models of cancer is the general animal—human translation problem [41]. This is a long-standing problem, which was considered specifically for the EMT6/Ro cell line as far back as Rockwell in 1980 [42]. As indicated above, the present study's translational efficacy is likely strongly tied to the interaction between the underlying cell-cycle (and phasing) characteristics of the cell line in question and the fractionated dose timing. With adequate characterisation of the former by standard methods, we believe that clinical translation is reasonably likely, especially given the relatively minor deviation our study would suggest away from standard multi-fractionated programs widely used in the clinic.

We conclude by noting several areas of potential for the present line of work, and the development of the model which supports it. First, we have exploited only one degree of freedom in our constrained search of protocol space, one could, for instance, follow [22] and conduct a parallel study which seeks to find a (quasi-)optimal allocation of a total dosage over multi-fractions, holding the inter-fraction time-gap constant. Or, one could allow *both* the *size* and *timing* of the fractions to vary; or one could introduce a chemo-therapy module (as in [16]) to study *combined* chemo- irradiation- therapy protocols. Obviously, apart from the fixed time-gap, variable fraction study, all other lines of inquiry increase again the size of the already substantial protocol-space, meaning on the one hand that search may take longer, but on the other, that the probability of finding an even greater performance benefit over existing benchmarks arguably increases. Second, and not insignificantly, our work considers pre-vasculature tumours as noted earlier. This is both a beneficial simplification and constraint on our work. A fruitful line of model development would be to add a vasculature development module coupled to a requisite tumour–tissue interaction module, leading to the ability to study larger (vascularturised) tumours, and equivalently the impact of any protocol on healthy tissue. Applying a cost-function for healthy tissue damage during the search phase of our approach would no doubt improve the clinical acceptability of any candidate protocol which departed significantly from currently used clinical benchmarks. Naturally, any extension along these lines would rely heavily on additional experimental data on the cell-line in question to provide critical validation and calibration points in the model's further development. As it stands, this study has already come close to the frontier of such data. No doubt the experimental community will mobilise around this call as computational models grow in sophistication, fidelity, and predictive and explanatory power.

## Materials and Methods

### The MCS Growth Model

The present work builds on our previously reported EMT6/Ro [11], [12] mouse breast cancer cell line multi-cellular spheroidal (MCS), fully scaled, quasi-2D, CA simulation model. The model has been developed with the consistent aim of achieving high, multi-dimensional, fidelity when compared to published, experimental EMT6/Ro studies. Prior to the present work, the model has passed several developmental milestones including bulk, saturation and internal tumour dynamics without irradiation [11], and the model's dynamic response to single-dose irradiation [12].

In [11] and [12] the basic spheroidal growth model was described and analysed hence we provide here a summary only. For convenience, we provide further details of the underlying model in the File S1. The model includes several key environmental and developmental components which serve to mimic the *in vitro* experimental conditions most commonly found in the literature and the characteristic biological features of MCSs.

The tumour cells exist within a 2-dimensional lattice whose boundaries are constantly replenished with glucose and oxygen nutrients at 5.5 mM and 0.28 mM, respectively [37], [38]. For computational efficiency and proliferative accuracy a *many-to-one* assumption is made by placing multiple (*N*) cells within each lattice site location. Given the cell packing density for EMT6/Ro cells (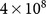
cell.cm^−3^, [37]) a lattice site side-length can be calculated. Thus, the choice of 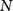 is the key scaling parameter of the model. As in our previous work, we set 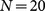 in the present study.

Diffusion of substrate nutrients (glucose and oxygen) and toxic metabolic by-products is achieved with a modified discrete diffusion algorithm (see [43] for details) to ensure an isotropic diffusion frontier [44]. The tumour mass is assumed to grow within a well mixed and constantly replenished media, hence, we assume constant substrate-concentration (Dirichlet) boundary conditions for glucose, oxygen and waste products (protons). At each update of the model (equal to 6 s) the diffusion module is run 24 times to ensure stability and accuracy as described in [12]. Diffusion coefficients for each of the three diffused quantities are set as described and explained in [12].

**Figure 5:**
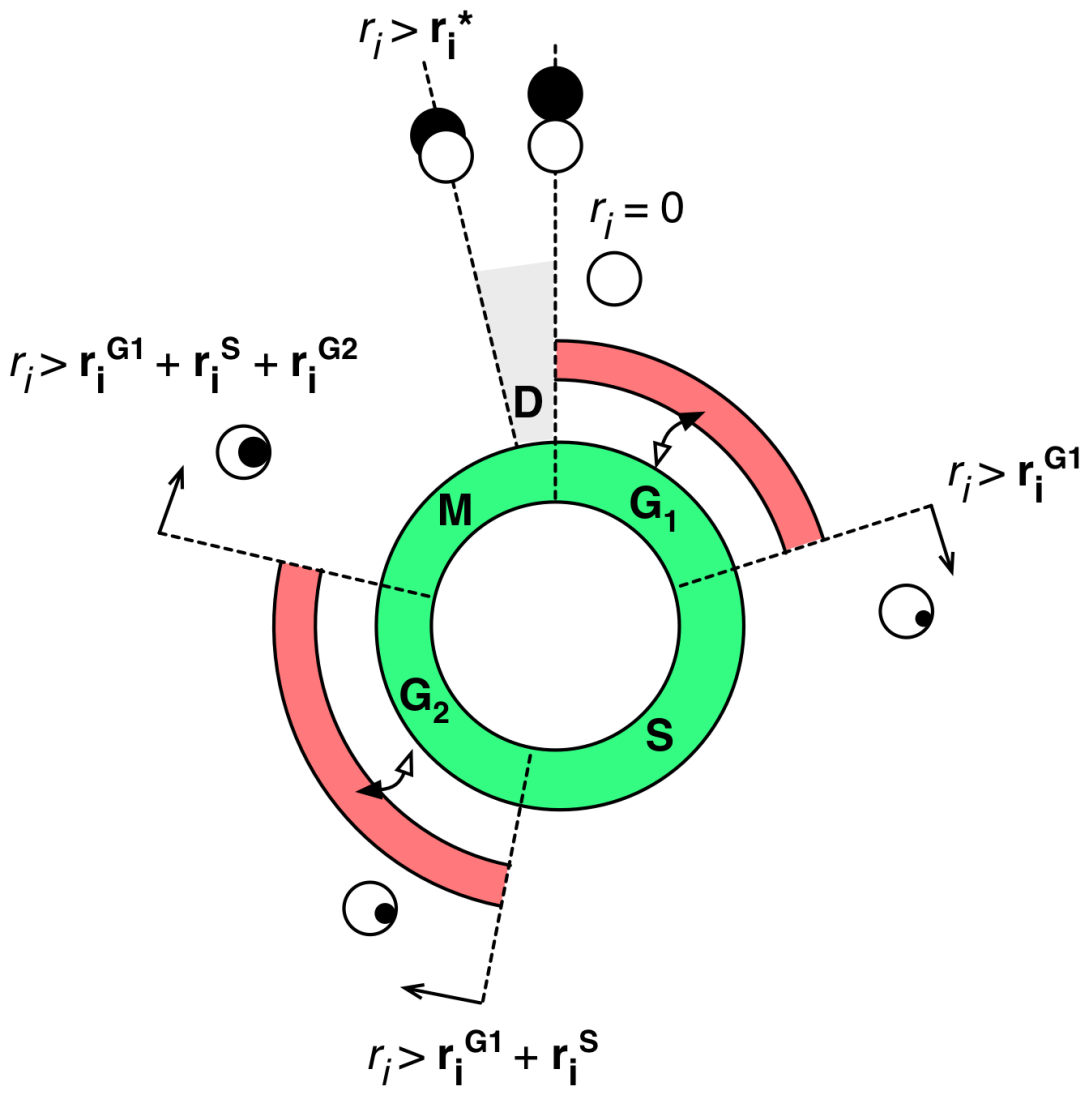
Representation of the cell cycle progression. Cells can be in one of the proliferative (green regions) cell cycle phases: 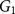, 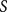, 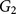 or 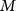. Each site is born with a unique combination of cell cycle phase timing durations (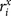, where 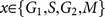). During the progression through the cell cycle the site cell cycle progression register is updated by 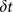 however only when the site can maintain a proliferative metabolism. It is assumed that due to the lack of nutrients or to low pH concentration cells can be forced to switch to the quiescence metabolism (red regions) pausing the progression through the cell cycle. Such a switch is assumed only possible in the 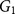 or 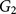 phases. Whenever a site enters the 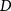 – division phase (which from the biological point of view is a part of the 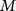 phase) and cannot manage to complete the division, the site death program is initiated.

Cell metabolism and cycling are replicated from [12]. Cells (sites) determine metabolism based on a multi-step decision algorithm which incorporates literature defined thresholds for known cell switching between proliferative and quiescent metabolisms in either aerobic or anaerobic modes for EMT6/Ro cells [27], [37], [39], [45]–[52]. (refer Fig.1(a) in [12]) When born, each filled site is endowed with cell cycle duration times, 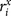 for each of four cycle phases (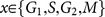) drawn from known normal distributions [53] for EMT6/Ro [27], summing to the sites total cell cycle duration, 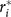 (Fig. 5). By default, each site is assumed to preference the proliferative (aerobic, if possible, or anaerobic, otherwise) metabolism mode, and so, progress around their cell cycle towards mitosis (refer Fig.1(b) in [12]). At each model update, the site cell cycle state impacts directly on the outcome of the cellular metabolism module since the latter depends on the former for biological reasons. For example, sites which have reached the (by definition) proliferative 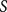 or 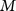 phases cannot access either the aerobic or anaerobic form of the quiescent metabolism. Failure to obtain enough nutrients for proliferation in these modes will enroll such a site into the site-death module. Both the cell cycle and metabolism algorithm modules are fully described in [12].

Sites which reach the final, division phase of their cell cycle have a fixed amount of time to finalise division (*D* phase, a sub-phase of the 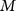 phase). Successful mitosis requires that a vacant site exists in the Moore (8-neighbour) neighbourhood of the parent in the lattice. Daughter sites are endowed with new draws for their cell cycle parameters from the distributions previously described. Both the parent and daughter cell cycle register is set to zero for subseqent progression around their respective cell cycles. The site death module leaves a vacant site and produces a one-off, toxic, necrotic material (see Table S5 of File S1).

Model timing is retained from [12] incorporating the updated two modules (*irradiation* and *repair*, described below): 1) Substrate replenishment; 2) Diffusion of all nutrients and toxic substances; 3) Irradiation of the tumour (if applicable); 4) Site metabolism decisions; 5) Nutrient metabolism; 6) Cell phase decisions; 7) Site repair or death after irradiation (if applicable); and 8) Cell division.

All parameters used in the present study are consistent with those reported in Table S5 of File S1. The *in silico* cultivated MCSs dynamic agrees with the tumour bulk properties experimentally measured and published in [37]–[39], [54], [55] including: the tumour growth rate based on the tumour volume and the diameter changes in time, the saturation cell count, the onset of necrosis, the thickness of the viable rim, the estimated doubling time, the saturation volume and the cell cycle distribution of cells within the tumour as a function of the tumour diameter, for details see [12].

### The Multi-dose Irradiation & Repair Module

To model accurately the tumour's response to multi-dose irradiation, we must extend our single-dose irradiation and repair module as described in [12]. Whilst our original repair module which included a cell-cycle delay proportional to dose and an increasing probability of cell death after attempted repair with dose are still applicable to this study we must make an important extension for the multi-dose setting. Specifically, the effective dose a site is subject to if it receives over-lapping irradiation fractions must be defined. Again, we turn to the experimental literature to define the multi-dose module:

First, we assume that DNA breaks (both single and double) are proportional to the dose of irradiation in use as it is shown for EMT6/Ro cells by Longo *et al.* [56] and in other cell lines (e.g. [57] for SC3T3/W and BALB cells).

Second, whilst repair of damage due to irradiation is widely supported in the experimental literature for EMT6/Ro cells [40], [47], [58]–[60] (see discussion in [12]), there has been some discussion over the functional form which best represents the repair process over time. We follow the conclusions of Fowler's detailed comparitive curve-fitting studies [17], [61] who has shown for several cell lines that a simple reciprocal repair function provides a better fit to the data than either a mono-, bi-, or multi- exponential process. Data presented in [57] (Fig.5) and [56] (Fig.4) show the same for EMT6/Ro cells specifically.

Third, we incorporate the important finding of Carabe-Fernandez *et al.* [62] who show by conducting a horse-race between a ‘full repair’ and an ‘unrepairable fraction’ model that the latter performs much better in fitting the experimental data. Indeed, of 16 experiments they analyse, 12 are better fitted (by F-test) by an ‘incomplete repair’ model. The finding is supported by Biedrmann's EMT6/Ro study [57] (Fig. 5) which shows that for a dose of 50 Gy, the EMT6/Ro cells repair only around 85% of the DNA breaks, with the authors noting that for some cell lines, the repairable fraction is much less than this. Indeed, Fowler in [17] emphasises (p.149) that for the spinal chord under multi-fraction (2 Gy) protocols, ‘the repairable fraction is half of the total damage’.

Fourth, data presented in [17] indicate that there is a single (global) half-time of repair (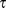) for a cell-line at a given temperature.

Taking these findings together, we develop a multi-irradiation module along the following lines:

1. For each site 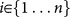 define by 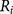 and 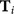 to be registers for site 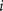 of the *current effective dose* (Gy) and *cell-cycle repair-delay* (h) respectively. These registers are both set initially to 0 and are only updated on the arrival of a new irradiation fraction at the site.

2. Let 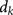 (Gy) be the dose of the 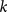
th irradiation fraction arriving at the site. Upon arrival of the 1st irradiation fraction of dose 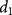 (Gy), all three registers are updated for the site: first set 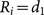; then, calculate 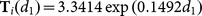 following our previously calibrated [12] repair-delay function for EMT6/Ro cells. Additionally, set a register for site 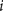 of time spent in repair, 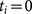 (hr).

3. Subsequent to the first fraction, any site which has reached a cell-phase boundary (refer Fig. 5) enters the repair module, and so, for these sites 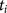 is incremented by model time to reflect the ongoing progress towards 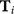.

4. Now, if no further irradiation fraction is received at site 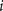, and the site has been in repair, if 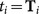, the site will undergo probabalistic death or be considered ‘repaired’ according to the single-dose irradiation module presented in [12]. However, where a site receives a subsequent irradiation fraction whilst either 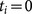 or 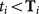, an update to the site's registers is made as follows: first, we assume that some fraction of the DNA breaks have been repaired since the last irradiation event, progress being measured by 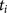 relative to 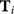, hence, calculate the equivalent remaining DNA break burden to be 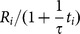 (following Fowler's reciprocal rule [17] where 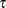 is a half time of reciprocal repair) and add the new damage burden (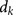) to this result. That is, update the register 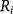,
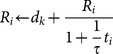



representing the total new level of DNA damage, after a history of partial repair, at a given site (which is the equivalent dose, Gy). Finally, update the site's other registers as in step 2. above.

Repair is assumed to take place for any site which has arrived at a cell-cycle boundary (e.g. G1/S, S/G2, or G2/M), hence, it is possible that a site which is only capable, for energetic reasons, of the quiescent metabolism and is in G1 phase, will undergo a step-function effective dose pattern since no repair time will have been accrued between fractions. Consequently, such a site will appear to have been ‘stopped’ in the given phase (e.g. G1, or G2). In aggregate, and over time, such a mechanism could lead to synchronisation of cell phases within the tumour mass [16].

### Calibration

In the reciprocal multi-dose irradiation repair module, 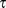 the half-time of reciprocal repair plays a vital role. For 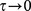 repair is extremely fast, causing the effective dose at a site to realise the long-run repaired level almost immediately, whilst for 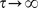 the rate of repair is effectively zero and causes the site effective dose time-series to approach that of the quiescent, non-repairing, site profile. Fowler [17] provides several estimates of 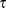 across different animal analogues: spinal cord cells in rats (2.8 h), mouse lung cells (0.9 h), pig skin cells (0.67 h), and rat foot cells (4.2 h), however, unfortunately no estimate is provided for EMT6/Ro cells – the cell line of the present study.

Given that cell line parameters cannot be interchanged [63] we calibrate our multi-dose irradiation module exclusively to available EMT6/Ro experimental data. Since we wish to study multi-dose protocols which utilise inter-fraction time delays on the scale of hours, we specifically focus on experimental studies which apply appreciably large inter-fraction delays. In this respect, the recent study of Otsuka *et al.* [18] and that of Sugie *et al*. [19] provide 12 and 6 independent protocol studies on the EMT6/Ro cell line, respectively. Together, they provide an extremely robust test of our multi-fraction irradiation module as they cover multi-dose experiments from two to five fractions, fractional doses from 4 Gy to 13 Gy, and inter-fraction delays from 15 min to 6 h.

In overview, our calibration strategy is to replicate the experimental conditions of both references and run each protocol over a range of 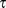 values for multiple synthetic spheroids, calculating for each 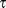 the final mean squared error (MSE) in mean effective dose to that reported in each reference. In our study, mean effective dose is equivalent to taking the average of 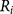 over all live cells at a given time point. The timing of the measurement is important due to repair processes undertaken by the repair module as outlined earlier. Hence, calibration proceeds by replicating the timing of the effective dose calculation of the experimental studies. To identify the optimal 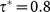 calibration value for EMT6/Ro cells, we find the 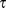 associated with the minimal MSE from both sets of experiments. In effect, treating each reference study as an independent calibration pole (full details of the calibration study are provided in the File S1).

### Search

#### Protocols, Protocol Space, & Benchmark Protocols

One can consider an *irradiation treatment protocol* as a vector of (dose, delay) pairs 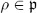,
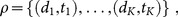
(1)where a given 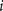
th pair defines the irradiation dose to apply at the present fraction (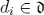, Gy) together with the wait-time between the previous fraction and the current one (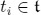, h). For example, a common irradiation protocol is to apply 2 Gy fractions each day of the working week (Monday–Friday). The first week of such a treatment program could be thus written,



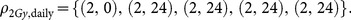
(2)The size of the total protocol space 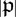, is then given by,
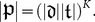
(3)


Obviously the granularity of 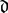 and 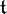 will impact non-trivially on 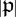. In this study, we focus on search near to clinically tested irradiation treatment protocols arguing that such *localised* search is likely to produce candidate protocols within clinically acceptable boundaries. Hence, we narrow our search to the vicinity of two particular protocols which mimic standard multi-fraction protocols in use in the clinic [5]–[7], and at that, we vary only the time-delay components (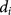) of these protocols, holding both the fractional dose and the total dose administered over the entire protocol constant (i.e. 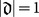 for all experiments). In Table 1 we give details of the two benchmark locations we shall be considering in the study (BMI and BMII), for each, we consider the first five days of a treatment protocol which may last several weeks.

To keep the size of 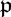 reasonable and to ensure that potential candidates are most likely to be clinically feasible we search in 30 min steps within a 22 h interval around the benchmark (see section Constraints for details).

#### The Case Library

Our aim is to discover protocols with *robust* performance over a diversity of tumours, avoiding over-fitting to a potentially pathological case. To accomplish this aim, a 10 tumour, 10 day, *case library*, 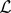 was developed in an identical manner to our approach in [12]: an initiating seed population of 200 cancer cells (10 sites) was placed *in-silico* in a well-mixed, constantly replenished substrate environment at 5.5 mM and 0.28 mM concentration of glucose and oxygen respectively (with pH maintained at 7.4 as in [12] and grown for 10 days before each tumour state was saved, and stored in 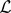 for later retrieval. Characteristics of 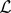 are given in the File S1.

#### Objective Score Definition

The objective of this work aligns with that of Engelhart's review of optimal chemotherapy treatment, to, ‘minimise the tumour size’ [23] (p.124). Specifically, we focus on the *number of tumour cells* existing at the end of each trial relative to the initial number of cells i.e. the normalized cell count. Our approach follows that of [16] in the context of the effectiveness of combined cancer therapies, or [64] which considers the effects of cell-cycle heterogeneity on the response of a solid tumour to chemotherapy, or [15] and [25] who study the effects of different radiotherapy schemes on tumour spheroids. Clearly, the volume of the spheroid could also be used (as is clinically common for practical reasons), however, tumour volume can hide significant changes which may have taken place within the tumour mass (e.g. increased necrotic radius) and so is a less informative summary measure of performance. Moreover, in case of the intense therapy it might happen that we observe the partial de- fragmentation of the tumour and then the measure of the radius of the tumour might be not relevant. Additionally, with *in silico* methods, number of cells is as computationally inexpensive as volume to calculate.

Prior to evaluating the candidate tumours in the rest of the study, the benchmark protocols were first evaluated, twenty times (20 random seeds), on each member of 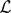. These data were then used as a baseline comparison to which the candidates could be compared by the fitness measure described below. Full details of the benchmark protocol performance on 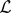 can be found in File S1.

Define by 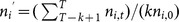 to be the *normalised cell count* for protocol 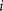, the ratio of the final cell count (averaged over the last 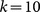 hours of experiment 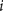 to account for any noise) to the initial cell count. Next, define by,
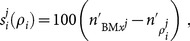
(4)the *fitness score* of protocol 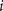 applied to tumour 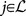, compared to benchmark BM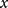 (where 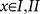). For example, a value of 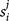 of 10.5 indicates that protocol 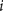 on tumour 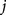 returned a 10.5% smaller cell-count at the end of the treatment period, relative to the benchmark protocol under study when applied to tumour 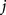.

Next, since we conclude that a protocol which outperforms the benchmark treatment a majority of the time, but performs substantially *worse* than the benchmark some of the time, does not represent a viable candidate for clinical application, we *bias* 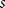 in the following way. In any situation where 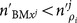, we apply the transformation 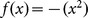 to the fitness score, effectively implying that when aggregating over all tumours in 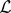, the protocol will suffer a severe fitness penalty.

The above approach yields a sample population of 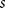 values, 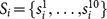 for some candidate protocol 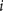, one for each 10 members of 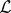, representing the expected performance of the given protocol on a diversity of case tumours. To rank each protocol, we finally construct a summary measure of the performance of the candidate by testing the hypothesis that the mean of the sample population is significantly greater than 0. That is, we construct, 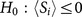; and alternatively 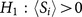, where 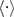 and 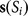 denotes the mean and standard deviation of the normal distribution 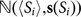 values respectively, and calculate 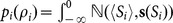. Ranking by 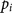 implies that for two candidates 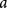 and 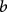, 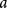 will be higher ranked than 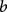 if 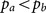.

#### Macro Search Technology: the Genetic Algorithm

The present search problem is well suited to discrete (or ‘*modular*’), parallel search methods: each solution comprises an ordered set of *delay* indices which point to the vector of feasible delay values. In such circumstances, continuous search methods are not feasible [65]. Moreover, since it is possible that small variations in the time-delay between given fractions may have a significant impact on the efficacy of a protocol (e.g. the issue of ‘missed days’ in treatment [66]), the solution landscape likely is non-linear in profile, again, challenging traditional search methods. However, genetic algorithms [13], [14] (GAs) are ideally suited to handling these solution contexts as they are naturally implemented with chromosome-like solution strings and handle well non-linear, modular, contributions to a solution's overall fitness.

GAs have been successfully applied across a vast number of scientific domains, with applications in radiotherapy beam-angle optimisation (for an overview see [2] or for examples [67]–[72]), radiotherapy patient flow planning [34], and applications in other areas of computational biology [35], [36] amongst them. However, to our knowledge, we are the first to apply GAs to the fractional dose program.

**Figure 6:**
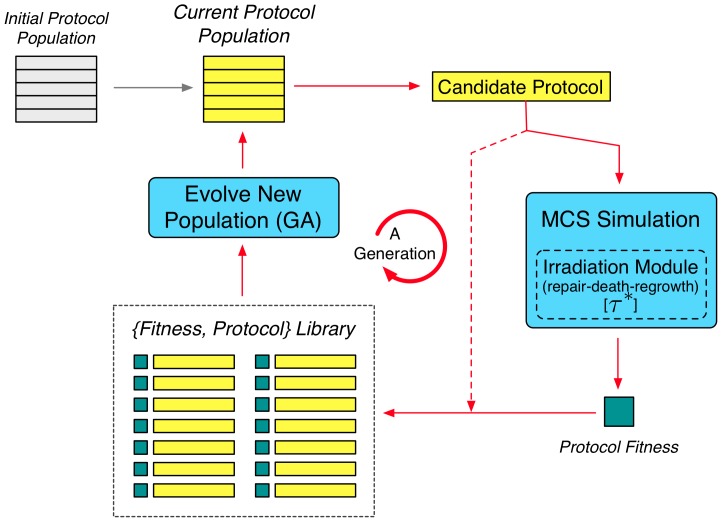
Representation of the Genetic Algorithm (GA) Search module. After initialisation, in each generation of the GA all candidates of the current population are run through the calibrated MCS and multi-dose irradiation model against all 10 case-library tumours, afterwhich the fitness (resultant normalised cell count relative to the given benchmark protocol) is calculated for each candidate before being added as a fitness–protocol pair to the GA Library. The new generation starts with the evolution of a new population by the GA module based on the full (updated) GA library. In the study over 1,100 candidates are evolved and tested in each benchmark. For a more detailed description of the whole procedure see File S1.

Figure 6 provides an overview of the GA approach. Each *generation*, a GA is applied to the growing library of *(fitness, treatment protocol)* pairs to produce a *population* of new protocol candidates for fitness testing. Prior to entering the main loop for the first time, the current candidate protocol population is initialised by random seeding. Subsequently, each generation proceeds in three phases: candidate testing; library addition; and new population formation:

1. Candidate Testing. During the testing phase, each candidate protocol in a generation is applied to each of the 10 tumours of the *tumour library* generating a vector of normalised cell counts which are used to compute an overall *fitness score* (4) for the candidate;

2. Library addition. Once all candidates in the current population have been tested, the population is added to the *candidate library* as (fitness pairs—candidate).

3. New population creation. The generation concludes when a new candidate protocol population is created via biased fitness selection and inheritence operators working on the entire (historical) protocol library. Practically, this means selecting one or two better performing protocols, ranked by fitness, from the library, and creating from them a novel candidate for testing by applying standard GA operators.

In broad terms, a GA takes a given ‘fitness’-ranked (highest objective score to lowest) population of solutions and uses these to produce a subsequent population of unique candidates via *biased inheritance*; the new solutions more likely possessing qualities found in the better performing solutions of the previous population (or ‘generation’). Figure 6 provides an overview of the GA employed in the present work. An initial population of 10 candidate protocols was created using random draws from the protocol-space. Full details of the GA including a description of the inheritance operators used are included in the File S1.

#### Constraints

The application of constraints to any search activity is critical: whilst the GA method could discover a given protocol which confers significant relative benefits, it will not be useful if the protocol leads to strongly negative side-effects (e.g. excessive normal tissue damage). For this reason, we apply constraints to our search with the central aim that any candidate protocol studied will depart only marginally from that already used in the clinic, reasoning that such a protocol will not only potentially confer therapeutic benefits but also retain a high probability of generating small, or at least, well-understood, side-effects for normal tissues.

First, since total dose delivered has material impacts on both the tumour and the healthy tissues [73], [74], we follow [34], [75] and constrain the total dose of any protocol to be 10 Gy in both benchmark environments (as per the benchmarks). Second, we apply lower, upper and granularity constraints to the inter-fraction time-gaps. Namely, for search in the vicinity of BMI and BMII, respectively, we define 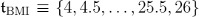
h and 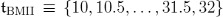
h. In each case, a 22 h time-gap interval is available, at 30 min steps. The interval is shifted lower in the case of BMI so that we can search the time-gap region below the smallest BMI time-gap of 6 h, whilst keeping the minimal time-gap distance appreciably greater than 0 so that we retain the focus on low dose, *fractionated* radiotherapy. Additionally, each chosen interval ensures that search goes beyond both solar (24 h) and cellular (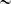 20 h for EMT6/Ro cells) cycles. The granularity choice of a 30 min step size is arrived at after considering: a) what could be clinically feasible (e.g. a protocol which relies on the fraction being delivered in a 5 min window is unlikely to be practical); b) the resultant size of the search space; and c) a small enough step size that cell phase cycle or metabolic cycles may come into play. Finally, in each benchmark study, the fractional dose is held constant at 1.25 Gy or 2.0 Gy respectively. Consequently, application of (3) implies search spaces for the two benchmarks of 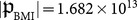 and 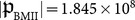 (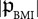: We have total dose 10 Gy, in 1.25 Gy fractions, implying at most 8 fractions; additionally, 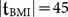; hence, to compute 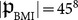).

To re-iterate, candidate protocols discovered by the GA will be identical to the benchmark in fractional-dose level, total-dose, and total treatment time; the only flexibility will be in the time-intervals between each fraction, within the given 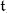 sets.

## Supporting Information

File S1 **This file contains supporting information on various aspects of the metholodolgy and results and is referred to in the main paper as ‘File S1’.**
(PDF)
