# Supplementary material for: A Matter of Timing: Identifying Significant Multi-Dose Radiotherapy Improvements by Numerical Simulation and Genetic Algorithm Search
Source: PLoS One. 2014 Dec 2;9(12):e114098. doi: 10.1371/journal.pone.0114098 (PMC4252029; doi:10.1371/journal.pone.0114098)
Supplement: File S1 — This file contains supporting information on various aspects of the metholodolgy and results and is referred to in the main paper as ‘File S1’. (PDF) [file pone.0114098.s001.pdf]

A matter of timing: identifying significant multi-dose radiotherapy improvements by numerical simulation and genetic algorithm search

Angus & Piotrowska, PLOS-One 2014

Supplementary Information

Contents

1 Calibration of the Multi-dose Irradiation & Repair Module: Details 1
1.1 Compilation of Reference Data . . . 1
1.2 The Calibration Assay . . . 2
1.3 The Optimal Value of τ . . . 4

2 Validation of the Model by Cell-Phase Dynamic Response to Irradiation 5

3 The Tumour Case Library: Details 6

4 Benchmark Protocols: Details 6

5 Objective Definition: Example 7

6 Search by the Genetic Algorithm: Details 8
6.1 The Inheritance Operators . . . 10
6.2 The Crossover Operator . . . 11
6.3 The Mutation Operator . . . 11
6.4 Constraints . . . 11
6.5 Performance . . . 11

7 Numerical Implementation: Details 11

8 The Model Lattice, sites & Metabolite Concentrations: Visualisation 12

9 Underlying Model: details 13
9.1 Numerical Diffusion . . . 13
9.2 The Metabolism Decision Algorithm . . . 13
9.3 Parameter Estimation . . . 13

1 Calibration of the Multi-dose Irradiation & Repair Module: Details

1.1 Compilation of Reference Data

We focus on experimental work which considers a matching cell-line (EMT6/Ro) and inter-fraction delay time-scale ($0-24$h) to our existing MCS model and clinically applicable time-scales, respectively. Two studies we are aware of fit these constraints, those of Otsuka *et al.* (2011) [1] and Sugie *et al.* (2006) [2]. Since both studies

arise from the same research laboratory – the Department of Radiology, Nagoya City University Graduate School of Medical Science – the methodologies and materials employed in each experiment are identical.

In the studies, around 2×10^5, exponentially growing, EMT6, cells were injected (transplanted) under the skin of female, 8-week old mice (prior to transplantation the cells were grown in Eagle's minimum essential medium with 12.5% fetal bovine serum). In both studies, the cells were allowed to grow to about 1 cm tumour diameter prior to the initiation of a given irradiation protocol. Of the protocols studied in the two papers, 12 experiments studied in the Otsuka *et al.* work [1] together with 6 experiments studied in the Sugie *et al.* work [2] are directly applicable to the present study.

In Table 1 of [1], calculated single dose equivalents (Gy) are given for multi-fraction experiments consisting of three dose per fraction levels for each of four total fraction count protocols. In all experiments in [1], each fraction was separated by a constant four hour inter-fraction delay.

Alternatively, in Sugie *et al.*'s work [2], Figure 1 and Table 1 in the reference present dose equivalent data on 6 experiments of interest, where two fractions of 10 Gy are given with varying inter-fraction delays of 15min to 6 hours. Whilst mean corresponding doses (Gy) are given directly in Table 1 in the reference, confidence intervals are reported only graphically (as one s.d.) in Figure 1 in the reference for *relative survival fraction* (SF). Nevertheless, it is relatively straight-forward to convert from relative survival fraction to dose equivalent via the survival fraction curve approach: noting first that on page 2 of the paper, the surviving fraction after the application of a single 20 Gy fraction was reported to be 0.010, the single parameter (for EMT6/Ro, $\alpha \sim 0$), β of the survival fraction model $SF = exp(\beta \times Dose)$ can be calculated to be -0.2303[1]; next, the relative SF s.d. bars can be located by simple data-grabbing software[2] and converted to survival fraction quantities by multiplying by the 20 Gy (baseline) figure of 0.010; finally, the error bar locations can be converted to equivalent dose by use of the SF formula, which leads to recovery of the s.d. quantum in equivalent dose form after differencing the mean equivalent dose (given in Table 1 of the reference).

Taken together, we compile 18 comparison experiments for calibration of the multi-irradiation model, given in aggregate form in Table **S1** below.

1.2 The Calibration Assay

Ideally, our MCS model would replicate the calibration experimental environment exactly. Since multi-fraction data are presently available only for *in vivo* mouse trials, and with relatively large (in *in silico* terms) tumours at that[3], a *direct* facsimile approach is not feasible *in silico*. However, the data are still highly useful to us since: (1) it has been shown that MSC *in vitro* studies reflect several important aspects of *in vivo* spheroidal conditions [4] and so can reasonaby provide relevant data for our computational spheroidal calibration; and (2) so long as essential features of the tumour morphology are replicated *in silico* (e.g. a necrotic core), calibration based on survival fraction assays already take into account scaling considerations.

Taking these factors into account, we replicate the calibration assay by applying the same irradiation protocols as the references to five *in silico* spheroids grown for the equivalent of 10 days from our computational *tumour library* (for details of the library, see section below). Whilst these tumours have a diameter of approximately 1 mm (a factor of 10 smaller than those implanted in the *in vivo* trials), the essential features of a growing, pre-angiogenic, spheroid mass are replicated (necrotic core, viable rim, cell phase distribution, growth and metabolism rates etc.).

For each of the 18 separate experimental conditions (see Table **S1**) five tumours from the library were re-initiated in the equivalent of 5.5 mM glucose and 0.28 mM oxygen concentration medium and irradiated according to the given protocols. After a total time of 20 h, counting from the beginning of the irradiation experiment (see p.20 of [2]), the experiment was stopped and the current effective dose level of each *in silico*

[1] The value of $\beta = -0.2303$ compares to the same calculated from data reported in [3] (Fig. 5, p. 890) of -0.2380.

[2] See Doke, J. (2007), '`grabit.m`', a MATLAB program to accurately obtain data from published images. Available from *Matlab Central*: `http://www.mathworks.com.au/matlabcentral/fileexchange/7173-grabit/content/grabit.m`.

[3] As per [1, 2] mice were implanted with around 2×10^5 cells, a number which, even with *many-to-one* scaling is at the boundary of feasible computational work.

Table S1: **Reference data available in the literature for calibration for multi-fraction experiments on EMT6/Ro cells.** Data complied from the studies of Otsuka *et al.* (2011) [1] and Sugie *et al.* (2006) [2]. Taken together, the studies provide 18 independant calibration markers. See text for details of data recovery.

Fraction Delay (h)	No. of Fractions	Dose per Fraction (Gy)	Dose Equivalent (Gy)	95% Conf. Interval
Data from Otsuka et al. (2011) [1]				
4	2	9	17.2	(16.0 - 18.5)
		11	20.4	(18.3 - 22.5)
		13	23.9	(21.6 - 26.1)
	3	7	19.0	(17.7 - 20.4)
		8	21.1	(19.4 - 23.1)
		9	21.5	(20.2 - 22.7)
	4	5	15.7	(13.5 - 18.0)
		6	18.7	(17.2 - 20.2*)
		7	23.9	(21.6 - 26.1)
	5	4	15.6	(14.8 - 16.4)
		5	20.5	(18.9 - 22.1)
		6	25.0	(22.9 - 27.1)
Data from Sugie et al. (2006) [2]				
0.25	2	10	20.4	(15.9 - 25.3)
0.50			19.0	(15.9 - 21.3)
1.00			20.0	(18.3 - 21.6)
2.00			18.6	(15.1 - 21.2)
4.00			18.3	(13.9 - 21.8)
6.00			17.2	(13.3 - 20.5)

* Note, Table 1 of [1] gives the upper bound of the 4×6 Gy multi-fraction study as '10.2', which we believe is a mis-print and should be '20.2' (as shown above): all other confidence intervals are symmetric around the mean, with 20.2 being the same distance above a mean of 18.7, as 17.2 is below.

cell (site) recorded (R_i, defined in the paper). For example, experiment one from Otsuka *et al.* [1] saw two, 9 Gy fractions applied to the tumour, separated by an inter-fraction delay of 4 h, and then, after a further 16 h, cell effective dose levels were recorded for analysis.

1.3 The Optimal Value of τ

According to the multi-dose irradiation and repair module definition (see paper) the multi-dose module has a single outstanding control parameter, τ, the half-time of repair for the EMT6/Ro cell line. To identify an optimal value of τ, each of the 18 separate multi-dose experiments reported in the reference studies were simulated, on all ten independent tumours from the tumour library (utilising a common random seed), over a set of 18 τ values in the set $\{0.1, 0.2, \ldots, 1.5, 1.6, 2.0, 4.0\}$ h (3,240 trials in all, 180 for each value of τ). The set of τ values scanned was informed by reported values for τ in Fowler [5] which range from 0.67 h (pig skin cells) to 4.2 h (rat foot cells).

In Fig. **S1** we present an example of the impact of τ on the final effective dose resulting from three different multi-dose irradiation protocols drawn from Otsuka *et al.* [1].

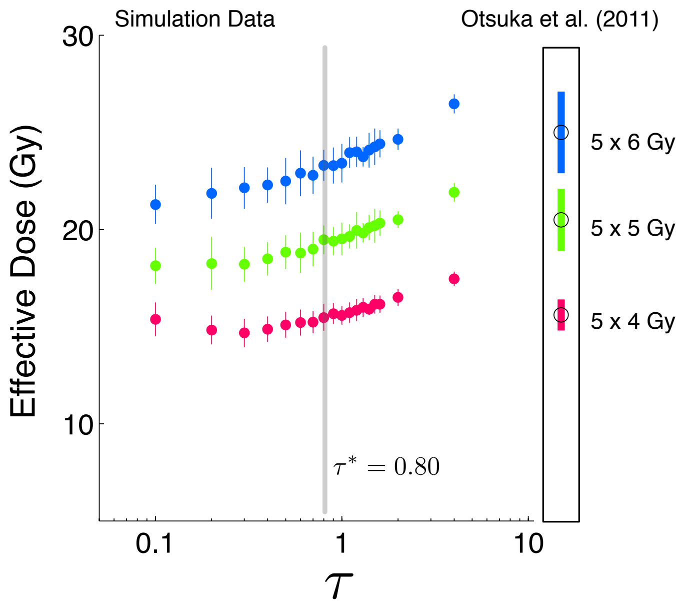

Figure S1: **Example impact of τ on effective dose.** Main panel: effective dose resulting from ten independent trials (ten different tumours) of a given multi-dose irradiation protocol at a given τ value, bars indicate $0.05 < p < 0.95$ confidence intervals; vertical grey line indicates location of optimal fitting τ^*. Right-panel: mean (open circle) and $0.05 < p < 0.95$ confidence intervals for experimental trials given in [1]. Multi-dose trials all comprised of five fractions at fractional dose as indicated, each with an inter-fraction delay of 4 h.

In all, the calibration simulations yielded 180 simulated values of mean effective dose,

$$R^{T,SIM} = (1/K) \sum_{k=1}^{K} R_k^{T,SIM} \quad (Gy),$$

at $T = 20h$ and for all active (filled) cells (sites) $k \in \{1, \ldots, K\}$ in a given trial. From these, the standard mean-squared error (MSE) formula was used to measure the distance from the simulated (SIM) values to those reported in the literature (LIT) (Table **S1**),

$$MSE_\tau = \frac{1}{M} \sum (R_m^{T,SIM,\tau} - R^{T,LIT,\tau})^2 \quad (Gy), \tag{1}$$

for all $m \in 1, \ldots, M$ replicates for a given τ value.

Figure **S2** presents the optimal fit surface in terms of the MSE associated with each of the 18 values of τ scanned in the simulations. The surface was found to be relatively smooth and single-peaked, leading to a choice of $\tau^* = 0.8$ which was subsequently used in the protocol search experiments in the paper. For a comparison of the effective dose observed by simulation compared with that of the experimental studies, see the figure in the paper.

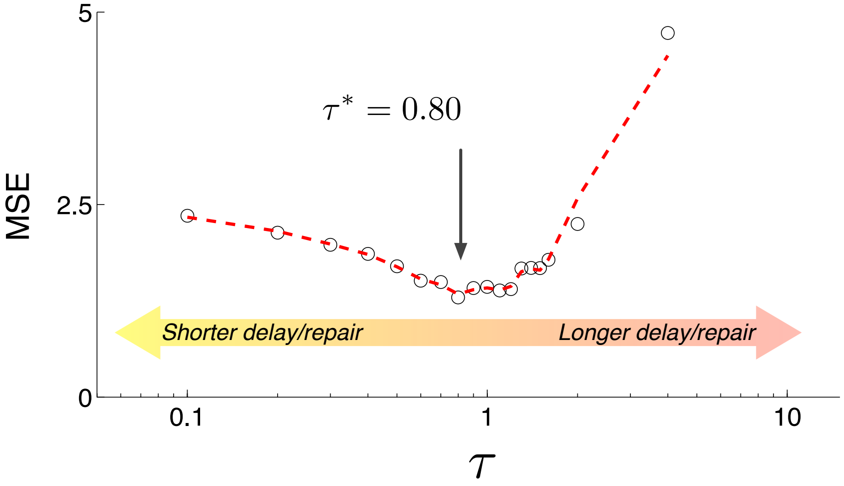

Figure S2: **Optimisation surface for half-life of repair (τ) calibration.** MSE values for the set of τ values scanned by numerical simulation of the MSC model under 18 different multi-fraction irradiation protocols reported in the literature. A simple smoothed trend line is shown (red broken line), along with the location of the optimal choice of τ revealed by the calibration study, $\tau^* = 0.80$.

2 Validation of the Model by Cell-Phase Dynamic Response to Irradiation

In our previous work [6] a calibration exercise was undertaken to match the bulk tumour growth dynamics under single dose irradition of various sizes to experimental data. In this work, having expanded the single-dose module to accommodate multi-dose irradiation, we have conducted (as detailed above, and in the main paper) a further calibration approach to the developed, multi-dose, model. As a further test of our model, and of importance to the present work which considers the impact of multi-dose irradiation on the dynamics of the cell-phase fractions, we wish to validate the cell-phase fraction dynamics of our model against experimental data. Unfortunately, we are not aware of any study which provides data of this type for EMT6/Ro cells. Nevertheless, we are aware of a single study which provides cell-phase fraction dynamics in response to *single* dose irradiation, namely that of Kal & Hahn (1976) [7].

In the reference, mice were innoculated with 100 mg to 400 mg tumours derived from 1.5 to 2×10^5 EMT6/Ro cells grown in cell suspension for two weeks, and were then exposed, *in vivo*, to a single dose of 300 rad (3 Gy) or 600 rad (6 Gy) irradiation, to a period of hyperthermia (43°C), or, to treatment consisting of a combination of the two administered in series. For our purposes, we focus on the cell-phase dynamics of the G_1, S and G_2+M cell fractions during the 28h after the single 600 rad treatment, as reported in Chart 7A of the reference. This chart presents the approximate results of the cell-phase dynamics for 'P-cells' (proliferative cells) only, the authors using data provided in Rockwell *et al.* [8] to calculate cell-fractions from the combined quiescent and proliferative population. The reason that we focus on Chart 7A is that in our *in silico* tumour, after an initial position of a roughly 1:1 ratio of P- to Q- (quiescent) metabolic phase cells, after irradiation, our computational tumour moves over the next 24-30h to an approximately 100% P-cell population. Further,

at this point, the *in silico* tumour begins to exhibit cell-phase dynamics consistent with the approximated P-cell figure of Chart 7 (see Fig. **S3**). Data were harvested from the reference using a standard, but precise

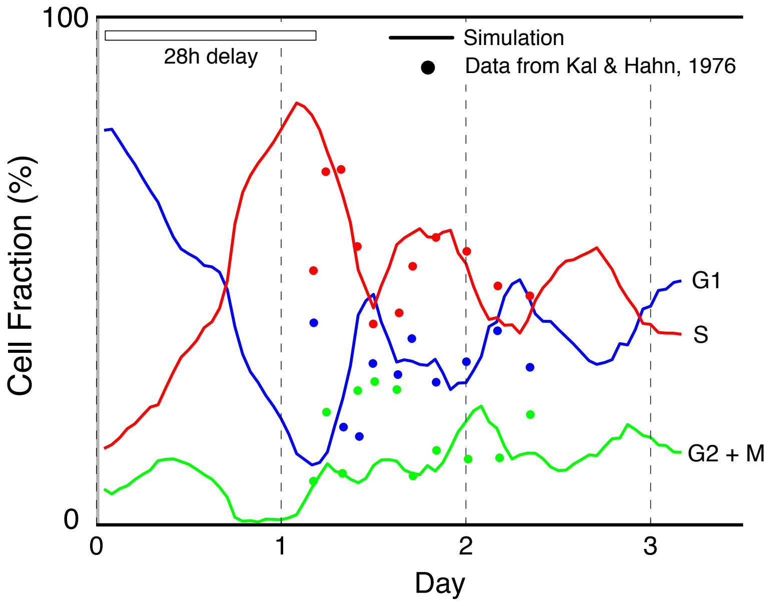

Figure S3: **A comparison of the dynamic response of cell-phase fractions to a single dose of 600 rad irradiation for the computational model described in the present study to that of an *in vivo* trial for the same cell line described in [7].** As indicated, experimental data from the reference have been shifted down-field 28 h to accommodate the simulation model's 28h transient period prior to establishing an approximately 100% P-cell metabolism.

data-extraction tool[4]. After the initial transient, the figure shows that the model provides quantiatively similar dynamic trajectories for each of the cell-phase groups under study.

3 The Tumour Case Library: Details

To evaluate systematically the efficacy of benchmark and candidate protocols we apply each protocol to a standardised *tumour case library*, $\mathcal{L}$. To construct the library, 10 scaled and calibrated synthetic tumours were initiated at day 0 with 200 cells and grown in the equivalent of 5.5 mM CHO and 0.28 mM oxygen media. Simulations followed the methods explained in detail in [6].

Table **S2** summarises the characteristics of $\mathcal{L}$, and provides some indicative characteristics from the available literature for EMT6/Ro cells.[5]

4 Benchmark Protocols: Details

In order that the candidate protocol efficacy could be evaluated, the control properties of the benchmark protocols were first measured. To do this, each benchmark protocol was applied 20 times (20 random seeds) to each of the 10 tumours in the relevant tumour library (i.e. 200 replicates in all).

Figure **S4** gives the normalised cell count trajectory for each benchmark protocol (with confidence interval). The figure clearly shows the periodic response of the tumour cell count to the applied irradiation fractions

[4]grabit.m, available from MATLAB's community code sharing website: `http://bit.ly/1kIKUpq`.

[5]For a direct (14 day, 20 day) comparison of the tumour model in use in this study to that of the available *in vitro* studies, please see the supplementary digital content associated with our earlier work [6].

Table S2: **Characteristics of the 10 day, 10 tumor, library used in the present study.** A comparison is provided to available *in vitro* literature for EMT6/Ro under equivalent medium conditions though over 14 and $\sim$ 20 days.

Measure	10 Day Library (for BM I, II)	*in vitro*† (at 14 or ∼20 days)	Units
Bulk & necrotic properties			
Final Tumor diameter	840 (±11)	est. 1050^a - 1250^c	μm
Diameter growth rate	77.3 (±0.4)	60^b - 79^a	μm/day
Diameter at onset of necrosis	638 (±28)	413^c	μm
Viable rim (post necrosis)	185 (±12)	207 $(\pm 13)^b$	μm
Gompertz fit properties			
Est. doubling time (vol.)	20.1	17^c - 18^d	h
Saturation volume	3.72×10^9	$6.3^c - 11.0^d \times 10^9$	μm^3
Saturation cell count	6.91×10^5	$7.0^c - 9.8^d \times 10^5$	
Cell phase fractions			
G_1	76.8 (±2.6)	60.1 $(\pm 5.3)^b$ - 76 $(\pm 3)^c$	%
S	14.8 (±3.0)	16 $(\pm 3)^c$ - 27.4 $(\pm 0.5)^a$	%
$G_2 + M$	8.5 (±1.9)	9 $(\pm 3)^c$ - 13.1 $(\pm 2.1)^b$	%

Notes:

† Experimental literature ranges given where available. Importantly, experimental values only available for tumors grown in the same media for 14 days or ~ 20 days as follows: 14 days {[a] [9], [b] [3]}, and $\sim$ 20 days { [c] [10], [d] [11], and [e] [12] }.

(indicated by blue lines in each panel). Additionally, the significance of the 5-day, post-treatment, period can be seen – the tumour re-growth at the cessation of treatment being a common feature.

To better visualise the impact of the irradiation fractions on the tumour cell phase kinetics, in Fig. **S5** we present the two benchmarks with their cell-phase fractions in time-series. Again, the strongly periodic influence of the regular fractions can be seen in the time-series. Of note, is that this periodicity extends well into the five day, post-treatment period.

Table S3: **Mean fitness score (final normalized cell count) as defined in the main paper for each of the two Benchmark protocols considered in the paper.**

Benchmark	$\langle s \rangle$	$(0.05 < p < 0.95)$
I	116.8	(95.7 - 134.7)
II	109.9	(91.6 - 128.3)

Finally, in Table **S3** we summarise the final normalised cell count of each benchmark, as defined in the paper. Against these numbers we will compare the candidate protocols which arise due to the genetic algorithm search procedure and construct the fitness score.

5 Objective Definition: Example

We provide here an example application of the calculation of f_i, the *performance* of candidate protocol i (refer to sub-section 'Objective Definition' in 'Materials & Methods' in the paper).

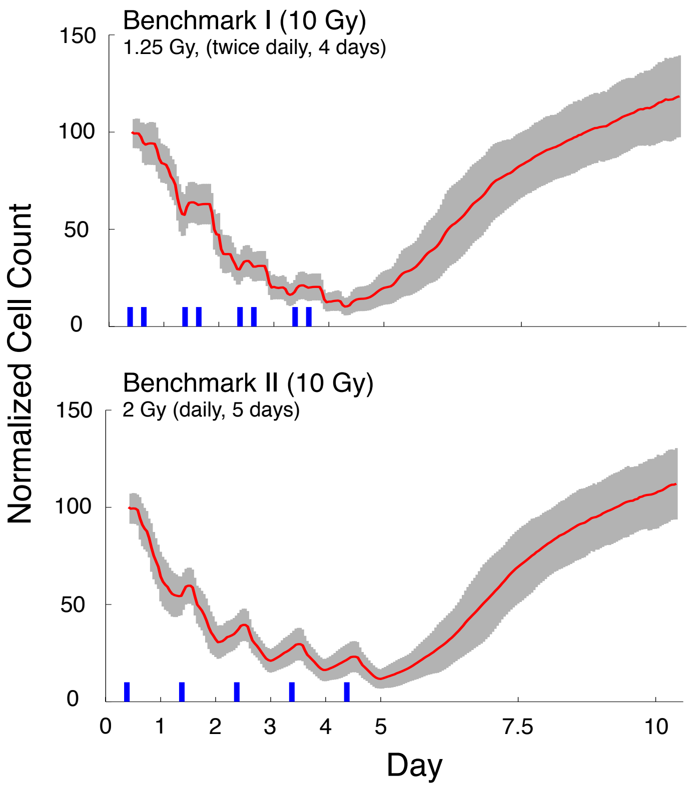

Figure S4: **Normalized (to day 0) cell count for benchmarks I and II as a function of time.** For benchmark I fractions of 1.25 Gy were administered twice daily during 4 days whilst for benchmark II fractions of 2 Gy were administered daily for 5 days. Note: the time axis has been shifted by 9h such that the first fraction occurs at 9.15am on the zeroth day of the trial. Fractional timing is given by the location of vertical blue lines. Means and $0.05 < p < 0.95$ confidence bounds are given by the red line and grey area, respectively.

Here, we use a genetic algorithm (GA) identified candidate, candidate `x1062` (which happens to be the best performing candidate from the benchmark I GA search). In Table **S4** and Fig. **S6** the data used to calculate the candidate's fitness score, $f_{\mathtt{x1062}}$ are given. First, a comparison is made between the normalised cell count of both the benchmark and the candidate for each tumour in the library (cols 2 and 3 of the table). Second, a relative score per tumour is derived, allowing the calculation of the probability that the candidate doesn't perform better than the benchmark. See section 'Objective Definition' of the paper for details.

Indeed, by applying the `normcdf` function from MATLAB, it is found, based on the sample population of relative scores in column 4 of Table **S4**, that zero (no improvement over the benchmark) would be a member of this sample with probability 0.62%. Or in other words, we can reject the null hypothesis that this candidate is no better than the benchmark with an expected judgement error of less than 1%. Note, the genetic algorithm search utilised only 1 (varying) random seed for each candidate. Hence, the apparent benefit of protocol 1062 would need further investigation with more random seeds to better estimate the likely expected benefit.

6 Search by the Genetic Algorithm: Details

Define by $G_n = \{\rho_1, \ldots, \rho_g\}$, *generation* n of irradiation treatment protocol candidates (as defined in the paper, see Materials & Methods section), and by $\mathfrak{L} = \{(s_1, \rho_1), \ldots, (s_l, \rho_l)\}$ a *library* of fitness—protocol pairs (s_i, ρ_i). Fittness—protocol pairs are added to $\mathfrak{L}$ after each generation is completed.

To produce a new generation, G_{n+1}, the genetic algorithm undertakes standard *biased inheritance* and

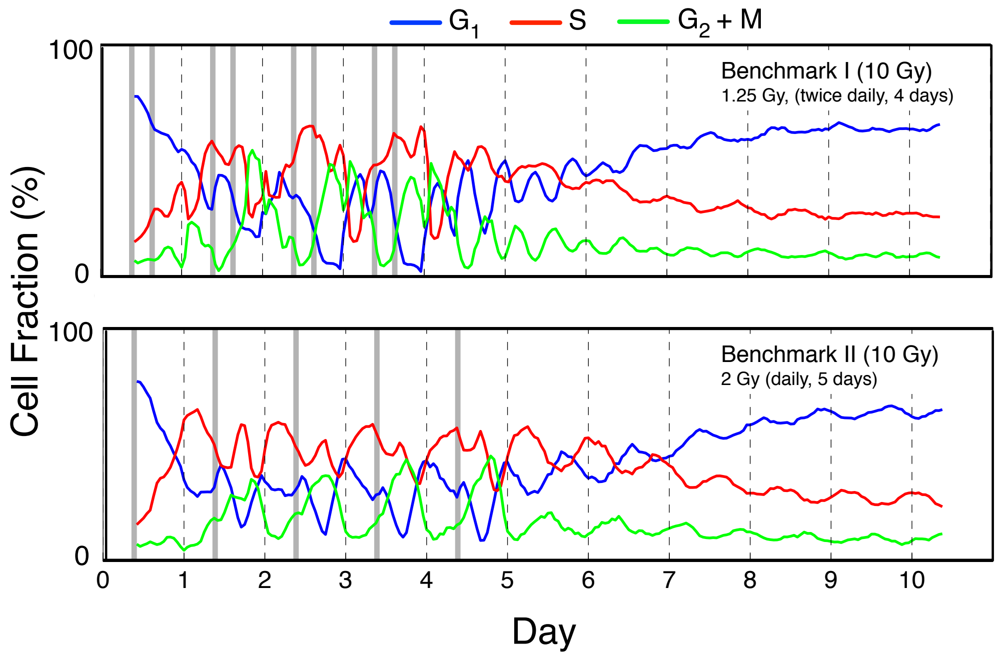

Figure S5: **Normalized cell fractions for benchmarks I and II as a function of time.** The mean cell fraction (given in percent) for tumours is shown for G_1 phase cells (blue line), G_2 phase cells (red line) and $G_2 + M$ phase cells (green line) over each of the 10 tumours in the tumour library and 20 independent random seeds (i.e. 200 replicates in all). The irradiation schemes and the substrate tumours are as for Fig. **S4**. Gray bars indicate the timing of irradiation fractions.

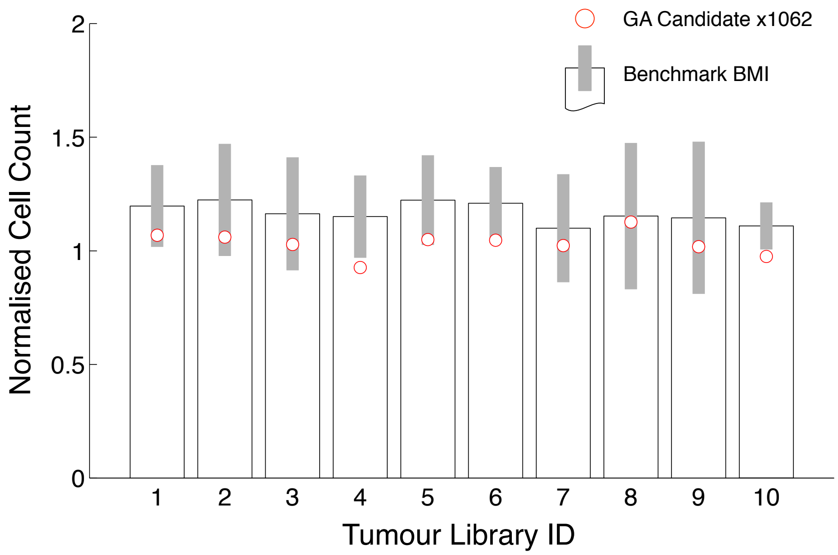

Figure S6: **Example comparison of normalised cell count between benchmark and GA identified candidate protocol.** Normalised cell count calculated for BMI protocol (n'^{BMI^j}) applied to each of the 10 tumours in the library (over 20 independent random seeds) versus the same for a genetic algorithm (GA) identified candidate protocol (protocol x1062, $n'^{\rho^j_{\mathrm{x1062}}}$). The difference between the normalised cell count over the 10 tumours is used to derive the overall fitness score, f_{x1062} of this protocol.

Table S4: **Example data used to derive the fitness score of a candidate.** Here, candidate x1062 is measured by normalised cell count against the same for the BMI protocol, allowing the calculation of relative score vector (col 3).

Tumor Library ID (j)	Norm. Cell Count		Relative Score
	Benchmark n'^{BMI^j}	Candidate x1062 $n'^{\rho^j_{\mathtt{ix1062}}}$	$s^j_{\mathtt{x1062}}$
1	1.20	1.07	13
2	1.22	1.06	16
3	1.16	1.03	13
4	1.15	0.93	22
5	1.22	1.05	17
6	1.21	1.05	16
7	1.10	1.02	8
8	1.15	1.13	2
9	1.15	1.02	13
10	1.11	0.97	14

novelty operators to produce novel candidate protocols based on the search space information held in $\mathfrak{L}$. By biased selection we mean that in each of the inheritance modules available, candidate protocols always inherit from higher fitness candidates in the library. The novelty operators provide a source of novelty to our new candidate protocols by changing, at low probability, the inherited protocol time-delay information. In addition, for three of the four inheritance operators (see below) a crossover operator is also used which mixes the protocol information from two higher performing protocols retrieved from $\mathfrak{L}$. In this way, the new candidate protocols are always developed based on better protocols already tried, with modification.

6.1 The Inheritance Operators

We utilise four inheritance operators in parallel. The first three operators yield two, better performing, parent protocols (ρ^*_i and ρ^*_j) to pass to the crossover operator. The final operator yields a single protocol only, which is passed directly to the mutation operator. The four operators are:

- TOURNAMENT: the TOURNAMENT operator randomly selects two candidates, i and j from $\mathfrak{L}$, considers their respective fitness scores s_i and s_j and returns the identity of the higher score candidate, splitting ties equiprobably. Consequently, the TOURNAMENT operator is the most broad in search scope of the four considered (it is feasible that the 2nd lowest ranked protocol is selected as a parent). The operator is run twice to yield two parent protocols which are passed to the crossover operator.

- TOP-DECILE: the TOP-DECILE operator first ranks all protocols in $\mathfrak{L}$ by fitness score and then, from the top 10%, randomly chooses two parent protocols to pass to the crossover operator.

- TOP-5: the TOP-5 operator works in the same way as the TOP-DECILE operator but instead of using the top 10% by fitness score, randomly chooses the two parents from just the top five protocols by score in the library to pass to the crossover operator. Naturally, the TOP-5 operator cannot be utilised until at least five protocols exist in $\mathfrak{L}$.

- TOP-1: the TOP-1 operator takes the highest ranked protocol in the library and passes this protocol, ρ^* directly to the mutation operator.

Taken in order, the four inheritance operators select parent protocols from $\mathfrak{L}$ with increasing focus on the highest ranked protocols. Or in other words, the search scope around the existing high performing protocols is either large (e.g. TOURNAMENT) or very small (e.g. TOP-1). The genetic algorithm uses one of the four inheritance operators by uniform random selection.

6.2 The Crossover Operator

With the exception of the TOP-1 operator, all other inheritance operators pass (ρ_i^*, ρ_j^*) to the crossover operator. The crossover operator randomly chooses a break-point from inter- (d, t) pair positions and constructs a new candidate protocol, ρ^* by meshing the protocol segment to the left of the break-point in ρ_i^* together with the protocol segment to the right of the break-point in ρ_j^*. For example, suppose that each protocol is constructed of 10 dose–delay pairs. Suppose further that the third break-point is supplied within the crossover operator, then,

$$\rho^* = \left\{(d_1^i, t_1^i), \ldots, (d_3^i, t_3^i), (d_4^j, t_4^j), \ldots, (d_{10}^j, t_{10}^j)\right\} .$$

6.3 The Mutation Operator

Finally, the candidate protocol ρ^* enters the mutation operator. Here, each element of ρ^* is considered independently and with probability m, will be replaced by a randomly selected element from the corresponding $\mathfrak{d}$ or $\mathfrak{t}$ vectors. Practially, since we only search $\mathfrak{t}$ in the vincinity of a benchmark protocol with $|\mathfrak{d}| = 1$, the mutation operator works only on the time-delay elements of ρ^*.

6.4 Constraints

Since, by selection of $\mathfrak{t}$ it is possible for a protocol vector ρ to contain a program of irradiation that would not conclude during the 5 day treatment period, a check is made prior to initiation of a generation to constrain protocol treatment lengths to the 5 day period. Practically, this means that trailing protocol instructions which occur after the 5 day cut-off are dropped from the protocol. Likewise, whilst it is not possible for a protocol to deliver more irradiation than the set total dose, it is possible for a protocol to deliver *less* (normally occuring when the 5-day time-constraint has material impact). We choose not to modify such protocols, preferring instead to allow the GA to preferentially select against them over time.

6.5 Performance

The performance of the genetic algorithm (GA) can be tracked over time by considering the fitness scores held in $\mathfrak{L}$. In Fig. **S7** we show the maximum, minimum and median performance of the top 10 candidates in $\mathfrak{L}$ (by fitness score, f_i) as $\mathfrak{L}$ grows from size 11 to 1,113.

7 Numerical Implementation: Details

The model was implemented, as in our previous work [13, 6], in the scientific computing language, MATLAB (R2013a or R2014a). All GA Search numerical simulations were conducted on the Australian Synchrotron high-performance computing cluster[6] utilising up to 15 parallel virtual nodes at any one time, each harnessing 10 CPUs, 1 GB of RAM and 1 GPU. Other experiments were conducted on the author's Mac Pro (16 GB) work-station utilising up to 20 simultaneous MATLAB threads distributed across 12 physical cores, usually via two virtual nodes comprising 10 threads each.

For GA search, each candidate protocol was allocated to a single node on the cluster, where the candidate was deployed and simulated against each one of the ten, 10 day, tumours residing in the tumour library, one core per tumour (utilising MATLAB's `parallel` toolbox). In this way, whilst a single, 240 h trial of one

[6] See `http://massive.org.au`.

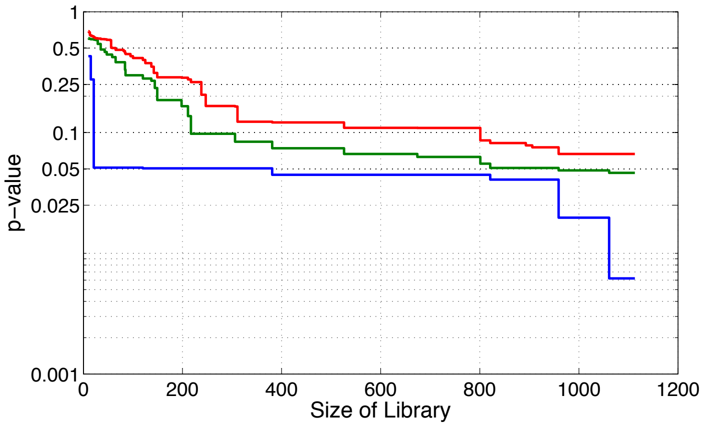

Figure S7: **Example of performance improvement obtained by GA search in the region of BMI.** The protocol–fitness library grows each time a new candidate protocol has been applied to the tumour and its fitness (f_i) recorded. As the library grows, we plot the p-value (one-tailed test that the normalised final cell count is smaller than the benchmark distribution) of the best (blue), median (green) and worst (red) of the top 10 performing candidates in the library.

candidate on one tumour took approximately 70 min of run-time to execute, in this time, all 10 trials on each tumour (each core) returned and submitted back to the common results library $\mathfrak{L}$, available simultaneously to all nodes. Standard locking of the library file was implemented to ensure that no read-write conflicts occurred during parallel processing. Taking together, with 13 virtual nodes in play, one 70 min period allowed for the analysis of 13 unique candidates over each of the 10 distinct members of the tumour library.

Taken together, the authors estimate that for all experiments reported in this work (calibration, validation, benchmarking, GA-search, detailed profiling of quasi-optimal candidates, periodic and periodic-deviation experiments) a total of 38,710 CPU h was utilised, or, under 4,000 hours of computation time on 10-core resourced machines.

8 The Model Lattice, sites & Metabolite Concentrations: Visualisation

In Figure **S8** we provide an example visualisation of a model run of tumour #5 in $\mathfrak{L}$ under the 17 h, perioidic, BMI $(8 \times 1.25\ Gy)$ environment. The first row of the figure demonstrates the initial setting of the tumour displaying the solid tumour periphery around a necrotic core. At this point, nutrient concentrations are well below the substrate level at the occupied sites, and remain low within the necrotic core, whilst waste-products (proxied as protons) have higher concentrations at the edge of the necrotic core, where cell-death is most likely.

The 2.5 and 5.0 day presentaitons demonstrate the impact of the irradiation schedule on the tumour mass and associated concentrations. Days 7.5 and 10.0 demonstrate the re-population period as surviving (repaired, sub-lethally dosed) sites re-populate the lattice demonstrating a degree of historicity as the new heterogeneous tumour mass develops.

In this figure, the (constant) substrate (surrounding) concentrations are represented by the maximum concentration ([CHO], [O_2]) or minimum concentration (H^+) as appropriate, normalised to the interval [0,1]. Substrate concentrations are held at 5.5 mM, 0.28 mM and pH 7.4 for glucose, oxygen and protons respectively. As a guide, the sequences of minimum site concentrations evident in each visualisation (or min pH for protons) from day 0 through day 2.5, 5.0, 7.5 and 10 as indicated for glucose, oxygen and pH were $\{0.28, 2.2, 2.8, 0.028, 0.0019\}$ mM, $\{8.9 \times 10^{-5}, 1.6 \times 10^{-3}, 1.3 \times 10^{-4}, 4.2 \times 10^{-5}, 2.0 \times 10^{-4}\}$ mM, and $\{6.71, 6.74, 6.81, 6.56, 6.59\}$ respectively.

The visualisation and treatment—re-population trajectory of Figure **S8** is reminiscent of the dose-painting

numerical simulation output given in Figure 8 of [14].

9 Underlying Model: details

In this section, we provide some further technical details on the underlying model upon which the present study builds. The underlying model is fully explained in [13] and [6], here we provide some further details for completeness in three areas: the approach to numerical diffusion, the metabolic decision algorithm, and the full parameter listing for the model with some commentary.

9.1 Numerical Diffusion

The tumour mass is assumed to sit within a 2D, connected, lattice, where each site is connected to its 8 Moore neighbours for the purposes of nutrient and waste transport, and cell division considerations.

The substrate (surrounding region beyond the growing tumour mass in the lattice) is assumed to be replenished periodically with oxygen (0.28 mM) and glucose (5.5 mM), and its pH maintained at 7.4. To account 2D lattice geometry approach for the diffusion of nutrients into the tumour mass from the surrounding substrate, and for the diffusion of waste matriels out of the tumour mass, we apply a slightly adjusted numerical diffusion equation.

Specifically, the concentration of each of glucose, oxygen, and waste x_i for $i \in \{CHO, O_2, H^+\}$ is computed for time $t+\tau$ based on the relevant concentration at time t at the site itself and at the site's surrounding 8 neighbours,

$$x_i^{t+\tau} = \frac{\alpha\beta\tau}{f}\left(\sum_{j\in\mathcal{O}^i} x_j^t + \frac{1}{\sqrt{2}}\sum_{j\in\mathcal{D}^i} x_j^t - f x_i^t\right) + x_i^t, \tag{2}$$

where $\mathcal{O}^i$ and $\mathcal{D}^i$ are the four sites orthogonal to, and adjacent to, the site of inquiry, i; α is the scaled diffusion coefficient for the given substance; β is a numerical correction factor for the spatial topology and τ is the time step. Equation 2 ensures that all nutrients and waste propogate isotropically to- and from- the tumour. Verification simulations (not shown) of the above formula show that it is identical on a 2D lattice with Moore neighbourhoods to the exact solution.

9.2 The Metabolism Decision Algorithm

Each model update, in addition to identifying a site's cell-phase (see Fig. 5 in the main paper), the site's metabolism mode is established. Figure **S9** provides the metabolism decision algorithm. Every update each site is considered using the algorithm, regardless of its previous metabolism state.

Initial decisions in the algorithm relate to sites which are unviable due to insufficient nutrients ($n_i < n^{q,ox}$), hyper-acidity ($pH_i < pH^{death}$) or if the site's metabolic clock has gone beyond its site defined total ($r_i > r^{max}$).

Next, if the site is in the G_1 phase, is near the G_1/S phase boundary (in metabolic time), but does not have sufficient free surrounding sites to place a viable daughter, the site cannot progress in proliferation and a quiescent metabolism is enforced (following the right-hand-most pathway in the figure).

For other sites, successive proliferative criteria are checked which determine the type of metabolic activity the site can sustain. The over-riding principle is that proliferation is preferred to quiescence. In the figure, the various critical (pH) and metabolic rates for aerobic and anaerobic versions of quiescent and proliferative metabolism can be found in Table **S5** below.

9.3 Parameter Estimation

The full set of paramters used in the study are presented in Table **S5**. The overall approach of the simulation exercise was to bring established quantities from the empirical literature, via scaling, directly into the numerical model. As can be seen in the the final column of the table, the mature experimental literature for EMT6/Ro

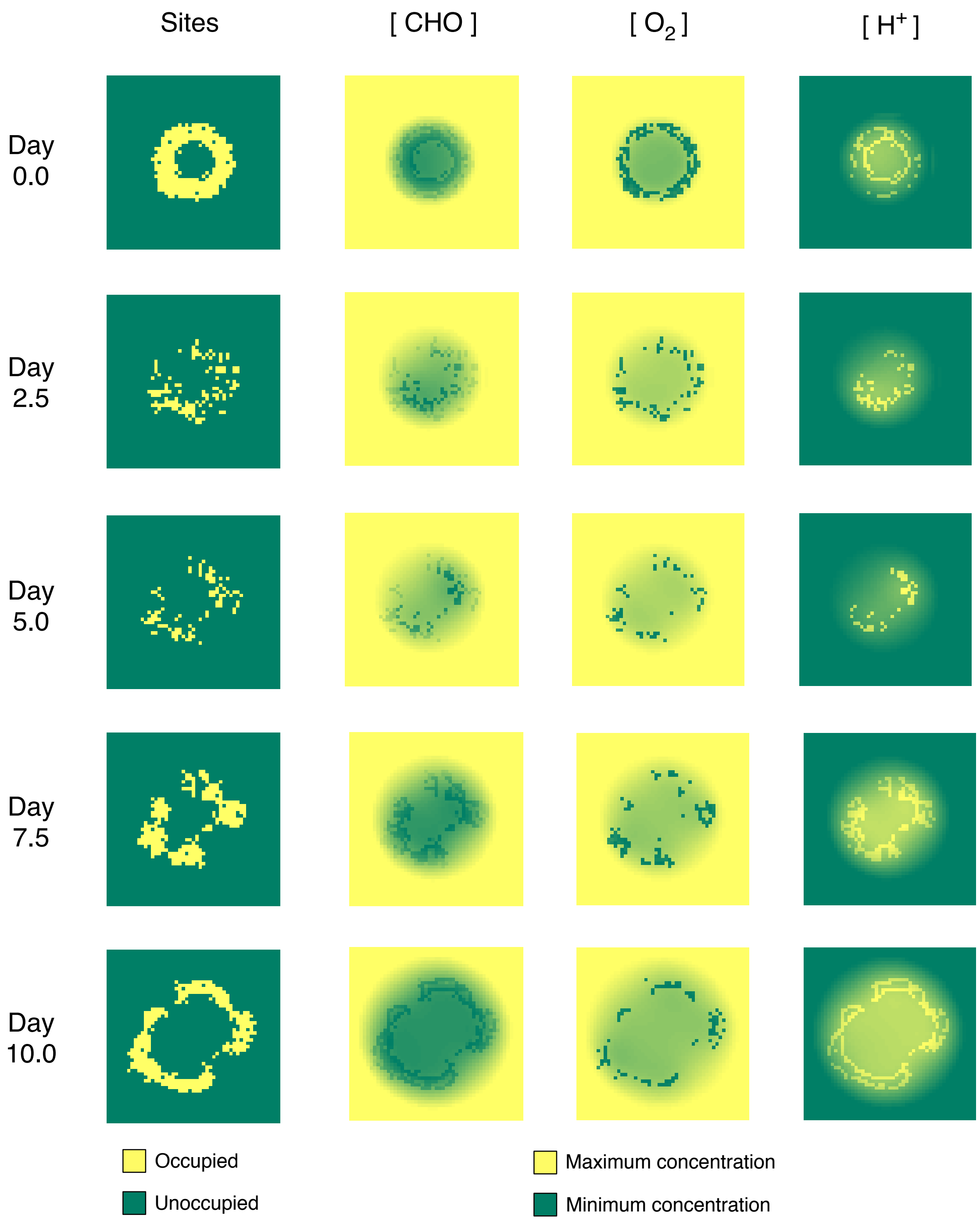

Figure S8: **Example spatial visualisation of the model under the 17 h, periodic, BMI protocol** The first column represents the location of occupied and unoccupied sites before (first row), during (rows 2 and 3), and after (rows 4 and 5) multi-fraction irradition. In this protocol, 1.25 Gy fractions are administered at $\{0.25, 17.25, ..., 102.25, 119.25\}h$. Columns 2, 3 and 4 represent the concentrations of glucose, oxygen, and waste products respectively at each given time.

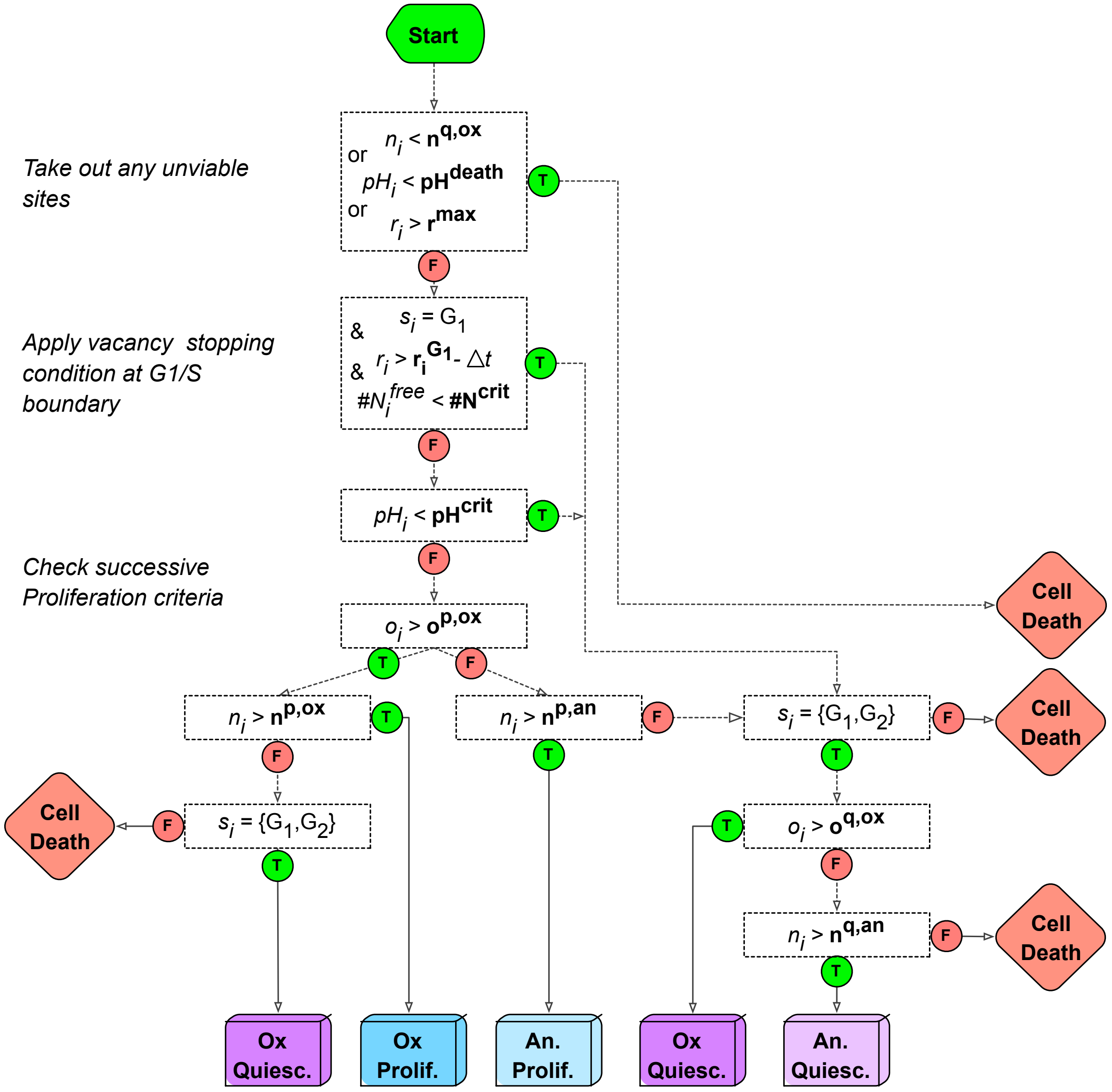

Figure S9: **The metabolism decision algorithm**

proved a facile path to parameter definition in the clear majority of cases. Where parameters were not available, estimates were made by the authors (indicated by 'est.' in the final column), based, where possible, on ralationships to other, known, parameters (see column 2).

Of note, the numerical model's bulk scaling properties are greatly influenced by the choice of: N, the number of cells per site; the temporal granularity paramters, δt and τ; and D_n, the glucose diffusion coefficient. The choice of N must balance the computational and other benefits of the *many-to-one* assumption (see [13] for discussion) and is the key bulk scaling parameter for the model (defining for example, the rate of growth of tumour diameter), whilst for our study, the joint choice of δt and τ had joint stability and dynamics-of-growth implications due to the numerical diffusion approach. In each case, we have opted for choices of these parameters that jointly: (a) are within the stability region of the numerical diffusion algorithm; (b) provide a match to the growth-dynamics of the EMT6/Ro cell line; and (c) that provide sufficient temporal granularity to resolve cell-metabolism and cell-phase switching.

Whilst the oxygen and H^+ diffusion coefficients were directly available from the literature (see Table **S5**), the literature is not settled on glucose diffusion coefficient value within the tumour mass ([15], [16], [17], and [18]). Indeed, estimates differ by several orders of magnitude. Hence, the glucose diffusion parameter was previously calibrated by fitting *in silico* experimental outcomes across both state and dynamic bulk and necrotic, Gompertz, and cell phase experimental data (for details see the Supplementary Information in [6]). The parameter used falls roughly in the middle of the reported, but varying, glucose diffusion coefficients available.

References

[1] Otsuka S, Shibamoto Y, Iwata H, Murata R, Sugie C, et al. (2011) Compatibility of the linear-quadratic formalism and biologically effective dose concept to high-dose-per-fraction irradiation in a murine tumor. Int J Radiat Oncol Biol Phys 81: 1538–1543.

[2] Sugie C, Shibamoto Y, Ito M, Ogino H, Miyamoto A, et al. (2006) Radiobiologic effect of intermittent radiation exposure in murine tumors. Int J Radiat Oncol Biol Phys 64: 619–624.

[3] Luk CK, Sutherland R (1987) Nutrient modification of proliferation and radiation response in EMT6/Ro spheroids. International Journal of Radiation Oncology*Biology*Physics 13: 885–895.

[4] Weaver VM, Petersen OW, Wang F (1997) Reversion of the Malignant Phenotype of Human Breast Cells in Three-Dimensional Culture and In Vivo by Integrin Blocking Antibodies. The Journal of cell biology 137: 231–245.

[5] Fowler J (2002) Repair between dose fractions: A simpler method of analyzing and reporting apparently biexponential repair. Radiat Res 158: 141–151.

[6] Angus SD, Piotrowska MJ (2013) A numerical model of EMT6/Ro spheroid dynamics under irradiation: Calibration and estimation of the underlying irradiation-induced cell survival probability. Journal of Theoretical Biology 320: 23–32.

[7] Kal HB, Hahn GM (1976) Kinetic Responses of Murine Sarcoma Cells to Radiation and Hyperthermia in Vivo and in Vitro. Cancer Research 36: 1923.

[8] Rockwell S, Kallman RF, Fajardo LF (1972) Characteristics of a serially transplanted mouse mammary tumor and its tissue-culture-adapted derivative. Journal of the National Cancer Institute 49: 735–749.

[9] Luk CK, Keng P, Sutherland R (1986) Radiation response of proliferating and quiescent subpopulations isolated from multicellular spheroids. British journal of cancer 54: 25–32.

Table S5: **Baseline model parameters with references.**

Description	Symbol	Value	Units	Reference[a]
Base model setting				
Cell packing density	ρ	4×10^8	cell.cm^{-3}	[10]
Number of cells per site	N	20	cell.site^{-1}	calib.
Unit side-length	u	38.8	μm	calc.
Update time-step	Δt	6	s	set
Diffusion time-step	τ	0.25	s	set
Cell cycle				
Av. G_1 phase dur. (s.d.)	$\bar{r}^{G_1}(\sigma_0^{G_1})$	$6(1)$	h	[19]
Av. S phase dur. (s.d.)	$\bar{r}^{S}(\sigma_0^{S})$	$10(2)$	h	[19]
Av. G_2 phase dur. (s.d.)	$\bar{r}^{G_2}(\sigma_0^{G_2})$	$2(0)$	h	[19]
Av. M phase dur. (s.d.)[b]	$\bar{r}^{M}(\sigma_0^{M})$	$2(0)$	h	[19]
Av. D phase dur. (s.d.)[b]	$\bar{r}^{D}(\sigma_0^{D})$	$0.1(0)$	h	est.
Maximal cell cycle time	r^{max}	20.1	h	est., [20, 10, 21]
Medium				
Medium [CHO]	n_{ex}	5.5	mM	[10, 3, 22]
Medium [O_2] conc.	o_{ex}	0.28	mM	[10, 3, 22]
Medium pH level	pH_{ex}	7.4		[10, 3, 22]
Critical pH *values*				
switch: prolif.→quiesc.	pH^{crit}	6.4		[23]
switch: quiesc.→death	pH^{death}	6.0		[24]
Diffusion coef.				
CHO diffusion coef.	D_n	9.5×10^{-6}	cm^2.s^{-1}	calib.
O_2 diffusion coef.	D_o	1.82×10^{-5}	cm^2.s^{-1}	[25]
H^+ diffusion coef.	D_w	1.1×10^{-5}	cm^2.s^{-1}	[17]
Proliferating Cells				
Aer. prol. CHO cons. rt.	$n^{p,ox}$	18×10^{-17}	mol.(cell.s)$^{-1}$	[10]
An. prol. CHO cons. rt.	$n^{p,an}$	52×10^{-17}	mol.(cell.s)$^{-1}$	[10]
Aer. prol. O_2 cons. rt.	$o^{p,ox}$	8.3×10^{-17}	mol.(cell.s)$^{-1}$	[10]
An. prol. O_2 cons. rt.	$o^{p,an}$	0	mol.(cell.s)$^{-1}$	[10]
Aer. prol. H^+ prod. rt.	$w^{p,ox}$	1×10^{-5}	mM.(s)$^{-1}$	[26]
An. prol. H^+ prod. rt	$w^{p,an} = 2n^{p,an}$	104×10^{-17}	mol.(cell.s)$^{-1}$	est.
Quiescent Cells				
Aer. quiesc. CHO cons. rt.	$n^{q,ox}$	15×10^{-17}	mol.(cell.s)$^{-1}$	[10]
An. quies. CHO cons. rt.	$n^{q,an} = \frac{n^{p,an}}{n^{p,ox}} n^{q,ox}$	43×10^{-17}	mol.(cell.s)$^{-1}$	est., [10]
Aer. quiesc. O_2 cons. rt.	$o^{q,ox}$	5.5×10^{-17}	mol.(cell.s)$^{-1}$	[10]
An. quiesc. O_2 cons. rt.	$o^{q,an}$	0	mol.(cell.s)$^{-1}$	[10]
Aer. quiesc. H^+ prod. rt.	$w^{q,ox}$	0.05×10^{-5}	mM.(s)$^{-1}$	[26]
An. quiesc. H^+ prod. rt.	$w^{q,an} = 2n^{q,an}$	86×10^{-17}	mol.(cell.s)$^{-1}$	est.
Dead Cells				
Dead cells CHO cons. rt.	n^{death}	0	mol.(cell.s)$^{-1}$	est.
Dead cells O_2 cons. rt.	o^{death}	0	mol.(cell.s)$^{-1}$	est.
Necrotic material prod.	w^n	9.0×10^{-4}	mM.(site)$^{-1}$	est.

Notes:
[a] 'calib.': parameter was defined by calibrating to emperical literature; 'est.': parameter and/or relationship assumed; and 'calc.': parameter the result of agebraic calculation of other parameters.
[b] the biological duration of M phase is given by $\bar{r}_M(\sigma_0^{r_M}) + \bar{r}_D(\sigma_0^{r_D})$.

[10] Freyer J, Sutherland R (1985) A reduction in the in situ rates of oxygen and glucose consumption of cells in EMT6/Ro spheroids during growth. Journal of Cellular Physiology 124: 516–524.

[11] Freyer J, Sutherland R (1986) Regulation of growth saturation and development of necrosis in EMT6/Ro multicellular spheroids by the glucose and oxygen supply. Cancer Research 46: 3504–3512.

[12] Freyer J, Sutherland R (1986) Proliferative and clonogenic heterogeneity of cells from EMT6/Ro multicellular spheroids induced by the glucose and oxygen supply. Cancer Research 46: 3513–3520.

[13] Piotrowska MJ, Angus SD (2009) A quantitative cellular automaton model of in vitro multicellular spheroid tumour growth. Journal of Theoretical Biology 258: 165–178.

[14] Alfonso JCL, Jagiella N, Núñez L, Herrero MA, Drasdo D (2014) Estimating Dose Painting Effects in Radiotherapy: A Mathematical Model. PLoS ONE 9: e89380.

[15] Freyer J, Sutherland R (1983) Determination of diffusion constants for metabolites in multicell tumor spheroids. Advances in Experimental Medicine and Biology 159: 463–475.

[16] Casciari JJ, Sotirchos SV, Sutherland RM (1988) Glucose Diffusivity in Multicellular Tumor Spheroids. Cancer Research 48: 3905–3909.

[17] Crone C, Levitt D (1984) Capillary permeability to small solutes. ML: American Physiological Society, Bethesda.

[18] Li C (1982) The glucose distribution in 9L rat brain multiceli tumor spheroids and its effect on cell necrosis. Cancer (Phila) 50: 2066–2073.

[19] Zacharaki E, Stamatakos GS, Nikita K, Uzunoglu N (2004) Simulating growth dynamics and radiation response of avascular tumour spheroids—model validation in the case of an EMT6/Ro multicellular spheroid. Computer Methods and Programs in Biomedicine 76: 193–206.

[20] Freyer J, Sutherland R (1980) Selective dissociation and characterization of cells from different regions of multicell tumor spheroids. Cancer Research 40(11): 3956–3965.

[21] Jiang Y, Pjesivac-Grbovic J, Cantrell C, Freyer J (2005) A multiscale model for avascular tumor growth. Biophysical Journal 89: 3884–3894.

[22] Kelley S, Kallman R, Rapacchietta D, Franko AJ (1981) The Effect of X-Irradiation On Cell Loss In Five Solid Murine Tumours, As Determined By the 125Iudr Method. Cell Proliferation 14: 611–624.

[23] Casciari J, Sotirchos S, Sutherland R (1992) Variation in tumor growth rates and metabolism with oxygen concentration, glucose concentration, and extracellular pH. Journal of Cellular Physiology 151: 386–394.

[24] Dairkee S, Deng S, Stampfer M, Waldman R, Smith H (1995) Selective cell culture of primary breast cancer. Cancer Research 35: 2516–2519.

[25] Venkatasubramanian R, Henson MA, Forbes NS (2006) Incorporating energy metabolism into a growth model of multicellular tumor spheroids. Journal of Theoretical Biology 242: 440–453.

[26] Patel A, Gawlinski E, Lemieux S, Gatenby R (2001) A cellular automaton model of early tumor growth and invasion: The effects of native tissue vascularity and increased anaerobic tumour metabolism. Journal of Theoretical Biology 213: 315–331.
